# DDCAL: Evenly Distributing Data into Low Variance Clusters Based on Iterative Feature Scaling

**DOI:** 10.1007/s00357-022-09428-6

**Published:** 2023-01-25

**Authors:** Marian Lux, Stefanie Rinderle-Ma

**Affiliations:** 1 Research Group Workflow Systems and Technology, University of Vienna, Vienna, Austria; 2 SWISDATA GmbH, Vienna, Austria; 3 TUM School of Computation, Information and Technology, Technical University of Munich, Munich, Germany

**Keywords:** Heuristic clustering, Classification, Data visualization, Choropleth maps, Process mining

## Abstract

This work studies the problem of clustering one-dimensional data points such that they are evenly distributed over a given number of low variance clusters. One application is the visualization of data on choropleth maps or on business process models, but without over-emphasizing outliers. This enables the detection and differentiation of smaller clusters. The problem is tackled based on a heuristic algorithm called DDCAL (1d distribution cluster algorithm) that is based on iterative feature scaling which generates stable results of clusters. The effectiveness of the DDCAL algorithm is shown based on 5 artificial data sets with different distributions and 4 real-world data sets reflecting different use cases. Moreover, the results from DDCAL, by using these data sets, are compared to 11 existing clustering algorithms. The application of the DDCAL algorithm is illustrated through the visualization of pandemic and population data on choropleth maps as well as process mining results on process models.

## Introduction

In 2021, the size of information that was “created, captured, copied, and consumed worldwide” accounted for 79 zettabytes and will grow to 181 zettabytes in 2025.^1^ “Information visualization can accelerate perception, provide insight and control, and harness this flood of valuable data to gain a competitive advantage in making business decisions” (Al-Kassab et al., 2014). One way to visualize information is to enrich an analysis model by assigning colors to different elements of the model where the basic idea originates from cartography (Coulson, 1987). An example for such analysis models is process models that describe the process logic of business processes, e.g., a patient treatment or manufacturing process. Then, for example, the service time or the frequency of an activity representing a node can be mapped to different colors (van der Aalst, 2016). In order to assign the colors, often clustering is employed as pre-processing step, i.e., the data is clustered and colors the area assigned to the resulting clusters (Jiang, 2013).

**Fig. 1:**
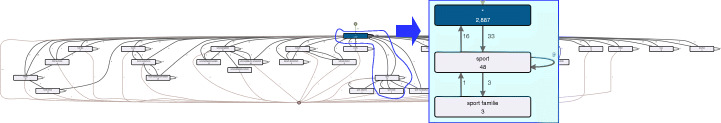
Mined process model using DISCO on Data Set 1 containing frequencies of activities (search terms)

Process models can be designed by domain experts or discovered from process execution data stored in process event logs, based on process mining techniques (van der Aalst, 2016). Process mining constitutes one of the key technologies for digital transformation (Reinkemeyer, 2022). One example use case is to mine process models from an information system, containing a keyword-based search functionality. Each search term appears several times in the event log and represents a node in the finally mined process model. The resulting process model is depicted in Fig. 1 by using the process mining software DISCO^2^ on Data Set 1 which is further described in Appendix 1.

The search term * is represented by one node in the discovered process model and is marked as the most frequent term in the process model with a frequency of 2.887. The second most frequent search term has a frequency of 90. Both frequencies 2.887 and 90 are unique for the data set. By contrast, 7 different search terms have a frequency of 3 and 6 different search terms have a frequency of 4 where the frequencies of 3 and 4 account for the lowest frequencies in the data set. For all 33 search terms, 17 different frequencies have been observed. Overall, the data set, as for many real-world applications, contains huge gaps between data points, i.e., the frequencies of the search terms. As depicted in Fig. 1, the process mining software DISCO maps the frequencies to colors of the process model nodes which range from dark blue to light gray. As stated in van der Aalst (2016), a process model can become more meaningful by mapping colors to its nodes. However, in this particular process model, the dominating search term * has such a high frequency that as a consequence, less frequent search terms appear all in the same color, i.e., light gray. This prevents differentiation between nodes such as “sport” with a frequency of 48 and “sport familie” with a frequency of 3. Therefore, not much information gain can be achieved through the usage of assigned colors.

Hence, it is of utmost importance to think about how to assign colors to the nodes in order to maximize the information gain of the visualization, even in the presence of “dominating” data points. Of particular interest is also the analysis of paths through the process model, including the happy flow of the process, which denotes the path taken by an average trace (Leemans et al., 2014) or more “exceptional” paths with particularly high/low frequencies of their nodes.

For the visualization of analysis models, often clustering is employed as pre-processing step (Jiang, 2013). Colors are then assigned to the clusters. If, for example in the process mining case (cf. Fig. 1), 10 colors are available for assignment to the frequencies and each frequency is assigned to one of these 10 colors, each color can be considered as a cluster. In order to meet these requirements, the clustering should be performed by considering a low variance inside each cluster and a wide distance between nearby clusters and at the same time an even distribution of data over all clusters. Without the latter, especially for real-world data sets, many clusters will be sparse due to outliers, tailed or non-uniform distributions. As a consequence, results can become uniform and colors assigned to clusters do not show much additional information when, e.g., investigating a particular path. However, there is a trade-off between a low variance inside each cluster with a wide distance of nearby clusters, and an even distribution of all clusters. This trade-off is investigated in this paper. In the following, possible approaches for tackling the problem are discussed.

At first, one could argue to classify the frequencies connected to nodes with unclassed colors. Doing so for each frequency a different color is assigned by using a color gradient which has the advantage of a “raw accuracy.” Exactly these discussions between unclassed and classed colors arose already in cartography when generating choropleth maps (Tobler, 1973), which is quite similar to the visualization problem as discussed here on process models. While unclassed colors have the advantage, as mentioned before, of a “raw accuracy,” classed colors are easier to process for humans. This is due to the few number of distinct colors to recognize, which helps to reduce the cognitive load by using a legend that lists the ranges of values corresponding to each color (Dobson, 1973; 1980). A naive pre-processing approach to generate classed colors would be to slice the frequencies into *m* equal intervals, where *m* is the targeted number of clusters. This approach is also used when creating histograms, where each class interval has the same width. While being simple and transparent, the approach might result in sparse or even empty classes, if the data set is not uniformly distributed or contains huge gaps, leading to show just two colors in extreme cases.

Another approach would be to use quantiles (*n*/*m*, where *n* is the number of data points) as classification method where the number of data points (aka frequencies) in each class is roughly equal, which overcomes the problem of equal intervals. However, this approach has many disadvantages. While it maximizes an even distribution of data into clusters, it does not consider any clustering by minimizing the variance inside each cluster and maximizing distances between clusters which may lead to problems of interpretation of colors in models. Another problem arises when there are many identical data points in the data set, which can lead to ambiguous classes.^3^

**Fig. 2:**
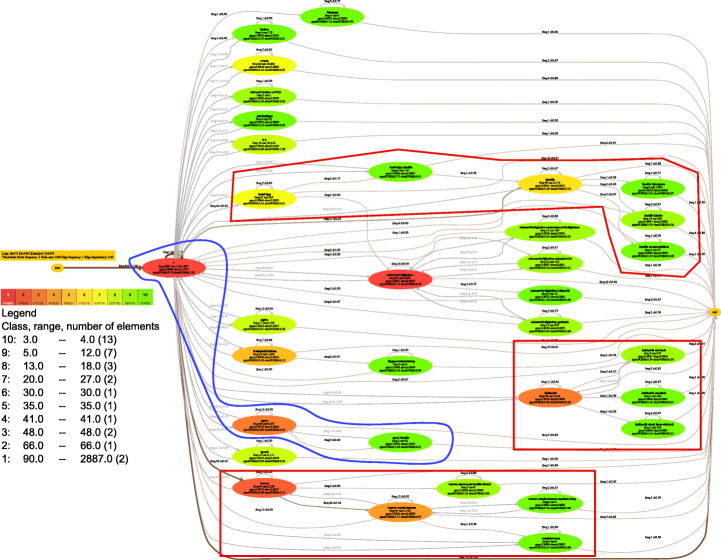
Mined process model from Data Set 1 containing frequencies of activities (search terms) assigned to colors through pre-processing with Jenks natural breaks

A more sophisticated approach on pre-processing, which comes from cartography and is well established there, is to use the algorithm called Jenks natural breaks, which produces a classification for a pre-defined number of classes that can be mapped to colors, by minimizing the variation within each class (Jenks, 1967). A result by using this algorithm on Data Set 1 is shown in Fig. 2. Here the blue frame shows the same activities as shown in Fig. 1, but in contrast, it becomes obvious that the three nodes in the process path indicate different frequencies.

The process model depicted in Fig. 2 conveys more information than the process model depicted in Fig. 1. Nevertheless, Jenks natural breaks is not optimized for considering an even distribution of the data points over all clusters, which helps to show differences between frequent elements in the process model, for example between frequencies 3 and 4. When discovering particular paths as shown in the red frames, such a distinction is useful to recognize differences in nodes and in further consequence, subpaths with splits at first glance. Also, a happy flow is hard to discover and “exceptional” paths with particular high/low frequencies of their nodes can be discovered, but not all related paths are highlighted in the process model. Another problem can be discovered from the legend in Fig. 2: Jenks natural breaks produces sparse classes (cf. classes with number 4–6, count only one data point), while putting the main outlier (i.e., the search term with a frequency 2.887) into the same class (cf. class with number 1) as the second most frequent search term (frequency 90) which both occurred just once in the whole process model.

Another use case is to divide students into equal learning groups in relation to an equal number of students and an equal level of knowledge in each group. Consider, for example, a previously executed test with 300 students, where 0–200 points were possible to reach. The test was performed at the beginning of a semester and the goal is to bring all students to a uniform level of knowledge. There are 6 teachers available to support the students during the semester, where each teacher teaches in one of 6 classes. Ideally, an equal number of students and an equal level of knowledge of students in each group is aspired. This problem can be solved by an approach that favors an even distribution in contrast to an equal level of knowledge.

In the following, the mentioned requirements and desired characteristics of the clustering algorithm for pre-processing to be designed and realized in this work are summarized. The algorithm is supposed to

**Table:** 

1. Handle one-dimensional data sets	
2. Handle negative and floating point data points	
3. Handle non-unique data points in data sets	
4. Define a targeted number of clusters (which can also be less), e.g., to map them with	
predefined colors	
5. Avoid overlapping cluster ranges	
6. Avoid filtering/omitting of elements by the cluster algorithm because it already	
performed through pre-processing steps like outlier detection if desired for a	
particular use case	
7. Reproduce the same clusters after each execution on the same data set, i.e., stable	
results of clusters	
8. Work on real-world data sets, e.g., data sets containing gaps between data points	
9. Produce fair results with classical clustering metrics such as low sum of variances	
(SV) from all clusters and a good score of mean silhouette coefficient (MSC)	
10. Result in an even distribution of data points into clusters	

The requirements and characteristics can be formulated into the following problem:

### **Problem 1**

Given a set D $\subseteq \mathbb {R}$ of (one-dimensional) data points and a targeted number of clusters *M* ≥ 1, where *m* is the number of actually built clusters, 1 ≤ *m* ≤ *M*.

Find clustering Cluster:$D \mapsto \mathcal {C} =<C_{1}, ..., C_{m}>$ with $C_{i}\subseteq D$, $\dot \bigcup _{i} C_{i} = D$ for *i* = 1,...,*m* with $\forall C_{i} \in \mathcal {C}$:

- |*C*_*i*_| converges to $\frac {|D|}{m}$ (Requirement 1) and ideally *M* clusters are built
- Minimize variance of *C*_*i*_ (Requirement 2)
- $\forall C_{j} \in \mathcal {C}, C_{i} \neq C_{j}:$ maximize distance between *C*_*i*_ and *C*_*j*_ (Requirement 3)
- Requirement 1 is prioritized over Requirement 2 and Requirement 3.

Problem 1 requires a given number of *M* clusters to be populated with data points under Requirements 1, 2, and 3. *M* is assumed to be determined based on the application, e.g., number of colors to be used for data visualization. 1 is measured with the metric SED (score even distribution) which is introduced in this paper. Similar to existing algorithms such as Jenks natural breaks (Jenks, 1967), Requirements 2 and 3 aim at finding low variance clusters which are separated from each other. And basically, cluster algorithms which produce low variance clusters do not intend to produce an even distribution of data points into clusters (Requirement 1), see Shapiro (2005). Unlike Jenks natural breaks, we do not consider the maximization of distances between the clusters (Requirement 3), in favor of achieving an equal distribution of the data points over the clusters (Requirement 1). This “forces” every cluster to be populated and avoiding the over-representation of frequent data or outliers at the same time. As discussed before, there might be a trade-off between Requirement 1 on the one side and Requirements 2 and 3 on the other side as filling all clusters might take a toll on cluster variance. This work addresses Problem 1 based on the following research questions:

1. How to design a (heuristic) clustering algorithm to evenly distribute 1d data points into a maximum number of low variance clusters?
2. On which underlying data distributions and real-world data sets does the algorithm perform most effectively?
3. How does the algorithm support data visualization?

To tackle RQ1–RQ3, this paper presents the heuristic 1d distribution cluster algorithm (DDCAL) that aims at balancing Requirements 1, 2, and 3 for Problem 1. The idea of DDCAL in a nutshell is to use an iterative approach by using the feature scaling method min-max normalization or also known as rescaling, for normalizing a sorted list of one-dimensional data points and to compare the results against defined boundaries from a set list. From each boundary, outliers from the upper or lower bound are considered as new cluster if the quantity of elements of a potential cluster is inside a given tolerance factor. Otherwise, the next boundary from the list is tested or if all boundaries were tested, the tolerance factor is increased and the testing of boundaries starts again. In other words, in every iteration step, the lower or upper quantity of outliers from a boundary, which is inside a given tolerance factor, that is used to support an even distribution of elements over all clusters, is chosen for building a new cluster.

In order to evaluate the effectiveness of the DDCAL algorithms and to compare them to existing algorithms, we use four quality metrics. We first analyze the results of the DDCAL algorithm in comparison to k-means++, Jenks natural breaks, head/tail breaks, DBSCAN, kernel density estimation (KDE), Gaussian mixture model (GMM), mean shift, largest gaps, Gaussian kernel k-means, k-medoids, and trimmed k-means on five artificial data sets, reflecting a selection of common distributions such as the normal and uniform distribution. The DDCAL algorithm is then compared to the before mentioned cluster algorithms based on 4 real-world data sets, i.e., process mining, weather, star distances, and population data, with respect to quality metrics. Finally, the applicability of the DDCAL algorithm for data visualization is demonstrated for process mining, US population, and Corona pandemic data sets. The real-world data sets were chosen because of their different data structures according to different distributions and different number of data points. Based on this, they illustrate the application possibilities of the DDCAL algorithm on different use cases.

Overall, DDCAL achieves better results than existing algorithms with respect to quality metrics and data visualizations on data sets with gaps, i.e., outliers, and tails. Moreover, the DDCAL algorithm yields promising results for data which contain just one peak with a distribution that looks like a bell curve (cf. normal/gumbel distribution). The results are even good if the number of peaks is equal or higher than *M*. The latter is reflected, for example, by the results for data with a uniform distribution.

The paper is structured as follows: Section 2 presents the DDCAL algorithm as well as the quality metrics. Section 3 comments on the implementation of the different algorithms and evaluates DDCAL on different data sets, which are real-world and synthetic data sets. The evaluation is compared with existing algorithms. Furthermore, different parameter setups on DDCAL are evaluated to demonstrate their implications on, e.g., different distributions. Section 4 shows applications from visualization of process mining results and maps. In Section 5, we discuss related work. Section 6 concludes the paper and discusses ongoing and future work. Appendix 1 details the data sets and Appendix 2 the implementation of the algorithms.

## 1D Distribution Cluster Algorithm (DDCAL)

This section provides the DDCAL algorithm, and metrics for assessing the quality of the clustering with respect to Problem 1.

### DDCAL

Given a set of 1d data points, the idea of DDCAL to tackle Problem 1 is as follows:

In the first step, the data points are sorted in ascending order. In a second step, the list of data points is copied and normalized by using the feature scaling method min-max normalization as described in Section 1. A list of boundaries to test outliers of clusters is defined. Boundaries from this list, starting with the lowest, are tested dynamically in each iteration where outliers from the upper and lower boundary are added to potential clusters. On these potential clusters, neighboring elements are added if the standard deviation inside the potential cluster decreases. If both potential clusters are above a set minimum quantity of elements — which is calculated from the combination of elements to even distribute over all remaining clusters and a set tolerance factor — the cluster with the least difference to the minimum quantity is actually built. If only one potential cluster or even no potential cluster is above the set minimum quantity of elements, the next boundary is tested and if all boundaries were tested without success of a new built cluster, the tolerance factor is increased and the set boundaries from the list, starting first with the lowest, are tested again as described before. Once a cluster is built, only the remaining empty clusters can be filled. The algorithm terminates if no empty cluster is left (i.e., of the maximum number of clusters M) or all data points are already assigned to clusters.

The pseudo code of DDCAL is provided in Algorithm 1 and explained in the following: The input data is an unsorted one-dimensional array of data points *d*∈ *D*. The output clustered_results is a list containing all data points from the input where each element consists of a data point *d* with an assigned cluster number. The algorithm has six parameters: (1) M contains the targeted number of clusters, e.g., 10 for 10 clusters to build. (2–3) boundary_min and boundary_max contain two parameters which define the minimum and maximum boundary for calculating outliers in each iteration to assign the data points from data to potential clusters. The parameters contain a value, ranging from > 0 to < 1, where, e.g., 0.1 stands for 10%, which means, that for a sorted normalized list of data points, outliers are the data points which are in the first 10% and in the last 10% of the list. (4) num_simulation defines the number of boundaries, ranging from boundary_min to boundary_max which are evenly spaced and stored in a list for finding the best boundary in each iteration step. (5) q_tolerance sets the quantity tolerance factor of elements for building a new cluster, which aims to produce only evenly distributed elements over all clusters within the defined factor. (6) q_tolerance_increase_step contains a growth factor which is responsible for increasing the q_tolerance factor, if all simulation steps for the list of different boundaries (cf. input parameters 2–3) did not satisfy the quantity tolerance factor.

**Algorithm 1:**
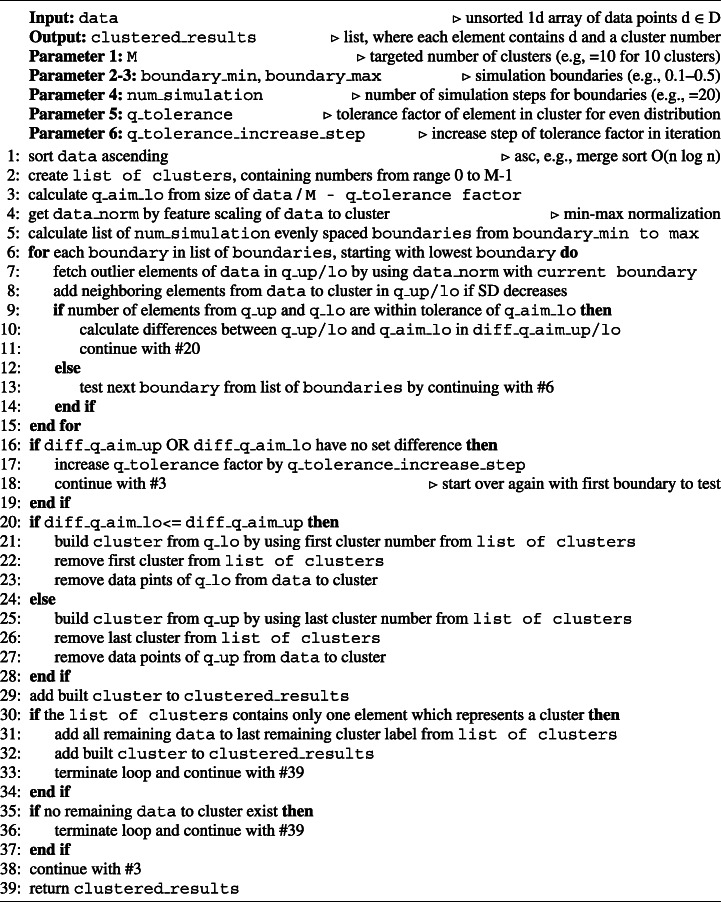
DDCAL (O(n log n).

As the first step (line #1), data is sorted in ascending order. In line #2, a list of clusters is generated from M, which contains M elements with ascending numbers, ranging from 0 to M-1. Next in #3, the temporary value q_aim_lo is calculated which determines the minimum quantity, a cluster must have to be created. The value is built by dividing the size of data which is not yet assigned to a cluster through the current q_tolerance factor. In the next step (#4), the data to cluster is normalized and stored as norm_data. For data points *D*, the feature scaling method min-max normalization is used with the following formula (Milligan and Cooper, 1988) to calculate relative results norm(*d*) of data point *d*∈ *D* depending on the respective minimum min(*D*) and maximum value max(*D*):

1 $$  \text{norm(\textit{d})}=\frac{\text{\textit{d}}-\min(D)}{\max(D)-\min(D)} $$

Continuing with step #5, a list containing num_simulation evenly spaces boundaries is created which comprises a sorted list of decimal numbers, ranging from boundary_min to boundary_max. From #6 to #15, boundaries from the list of boundaries, which was created in #5 and starting with the lowest boundary (i.e., first element in the list), are treated. On step #7, all outlier elements from data are determined by using the current boundary from the list of boundaries and comparing it with norm_data which contains the same data points on the same position as data. The lower outliers are stored in q_lo and the upper outliers are stored in q_up. Step #8 adds neighboring elements from q_lo/up from not yet added data, as long as the standard deviation inside q_lo/up decreases. From line #9 to #14, there is a check with handling defined, if the number of elements from q_up and q_lo is above q_aim_lo. If yes, the differences from the number of elements from q_up/lo and q_aim_lo are calculated and stored in diff_q_aim_up/lo and the algorithm continues with line #19. If no, a next current boundary will be tested by continuing with line #6. The lines #16–#19 are only reached if no boundary was found (#6–#15) which produced both, diff_q_aim_lo/up. Thus, diff_q_aim_up or diff_q_aim_lo is not set and the q_tolerance factor is increased by the factor of q_tolerance_increase_step (#17). The algorithm continues with line #3 by calculating the q_aim_lo again for all boundaries to test. In steps #20–#28, a cluster is built from the outliers q_lo or q_up. q_lo is chosen, if the associated diff_q_aim_lo is smaller or equal diff_q_aim_up. Then, on line #21, a cluster is built from the data points of q_lo where each data point is assigned with the first element from the list of clusters. Next, the first element is removed from the list of clusters (#22) and the data points from q_lo are removed from data (#23). Otherwise, if the diff_q_aim_up is smaller than diff_q_aim_lo, a cluster is built from the data points of q_up where each data point is assigned with the last element from the list of clusters (#25). Then, the last element is removed from the list of clusters (#26) and the data points from q_up are removed as well from data (#27). In both cases, the build cluster is added to the list of clustered_results (#29). Line #30–#34 handles if the list of clusters contains only one element. Then, a cluster is built with all remaining data points from data which are assigned to the element from the list of clusters (#31). Next on steps #32–#33, the cluster is added to the list of clustered_results and the algorithm continues with step #39. If there exist no remaining data to cluster, the algorithm continues as well with step #39 (#35–#37). Otherwise, after a new cluster was built and added to the list of clustered_results and there exists data to cluster and as well a list of clusters containing more than one element, the algorithm starts the next iteration for building a cluster and continues with step #3 (#38). Finally, on #39, the built list of clustered_results is returned as output of the algorithm.

DDCAL produces stable results, i.e., the output of the algorithm remains the same after each execution if the data set and the parameters do not change. Furthermore, the algorithm does not contain nested loops of the input list data which results basically in *O*(*n*) with *n* = |*D*| in terms of runtime or space requirements if the number of clusters is considered as constant and the sorting step (#1) is left out, where all general sorting functions are, at best, O(n log n).

The algorithm uses the feature scaling method min-max normalization with boundaries in an iterative approach. The concept is inspired from statistical tests based on data distributions and their significance threshold which is in the case of this algorithm defined through a boundary. DDCAL compares in every iteration the whole remaining data set for building a new cluster. The term remaining means in this case that data points are assigned to a cluster, which are typically outliers, and then these data points are removed from the data set of data to cluster. By addressing Problem 1 (cf. Section 1), DDCAL aims to increase the number of elements in each cluster which is reflected in evenly distributed elements over all clusters (cf. Requirement 1). The even distribution can be influenced with the parameter q_tolerance, which sets the minimum amount of elements for building a cluster on a given range of boundaries to test. And if these boundaries do not produce the minimum amount of elements as outliers, the parameter q_tolerance_increase_step increases the q_tolerance factor and thus, influences the even distribution of the results as well.

By using this iterative approach on the remaining data for building clusters with outliers, it becomes more likely to be able to add more data points to clusters in subsequent iteration steps on lower boundaries, because they contain less outliers. To add too many data points into a cluster is hampered through the logic, that on each iteration, clusters are built starting with the lowest boundary to test and a cluster can actually be built, if the minimum amount of elements on both sides, the upper and lower bound, are reached or exceeded where the side with the fewer data points is used for building a cluster. This benefits of course Requirement 1, but through the iterative approach of removing outliers in every iteration, the variance of the whole remaining data set decreases. Therefore, clusters with outliers have lower variances in their clusters in later iterations which benefits Requirement 2 and also indirectly Requirement 3.

### Clustering Quality Metrics

We use four quality metrics, i.e., number of used clusters (NUC), sum even distribution (SED), sum variances (SV), and mean silhouette coefficient (MSC) that capture the coverage of a clustering result with respect to Problem 1.

How evenly the data is distributed over built clusters is measured with the metric SED. The aim of DDCAL, which is described in detail in Algorithm 1, is to produce clusters with high SED values (Requirement 1), as top priority in addressing Problem 1, but also to consider Requirements 2 and 3 which are measured through the quality metrics SV and MSC. Section 3.4 employs the four quality metrics in order to compare DDCAL with other clustering algorithms.

Note, while DDCAL produces high SED values, we performed different simulations of parameters with existing algorithms if available and picked the results with the highest SED values in order to foster a fair comparison.

#### **Definition 1** (Quality Metrics)

Consider Problem 1 with D $\subseteq \mathbb {R}$ being a set of (one-dimensional) data points. Then, quality metrics NUC, SED, SV, and MSC measure the quality of a clustering *C* =< *C*_1_,...,*C*_*m*_ > (*m* ≤ *M*), where *M* denotes the targeted number of clusters. With respect to Requirements 1 and 2, the following quality metrics are defined:

- NUC := $\frac {m}{M}$
- SED := ${\prod }_{i=1}^{m} \mid C_{i} \mid $
- SV := ${\sum }_{i=1}^{m}{\sum }_{j=1}^{\mid C_{i} \mid } \mid \mid x_{ij} - z_{i} \mid \mid $ where *m* is the number of the actually built clusters, *x*_*ij*_ denotes the *j* th data point in cluster *C*_*i*_, and *z*_*i*_ the mean value of cluster *C*_*i*_ (see (Faber, 1994)).
- MSC : = $\frac {1}{\mid D \mid } * {\sum }_{d \in D} s(d)$ where *s*(*d*) denotes the silhouette coefficient for data point *d* ∈ *D* with $s(d)=\frac {b(d)-a(d)}{\max \limits \{(a(d),b(d))\}}$ where *a*(*d*) is the average dissimilarity of *d* to all other objects of *C*_*i*_, *b*(*d*) is the minimum *dis*(*d*,*C*_*j*_) with *i*≠*j*, and *dis*(*d*,*C*_*j*_) is the average dissimilarity of *d* to all objects of *C*_*j*_ (see (Rousseeuw, 1987)).

NUC ∈ [0;1] assesses the number of used clusters, in particular the implicit requirement to fill *M* clusters. NUC= 1 denotes the best result and NUC= 0 the worst. When comparing the different algorithms as described in Section 3, we compare just results with NUC= 1 which indicates that all targeted clusters were actually built and a fair comparison with the other clustering quality metrics is therefore valid. SED, the score of even distribution, evaluates how evenly the data is distributed over the clusters (↦ Requirement 1); the product of the cluster cardinalities is maximized for clusters of equal size and if NUC= 1. It is minimized for clusters, where every cluster contains just one element with the exception of one cluster which contains all remaining elements of a data set. Higher results of are better, where the best result appears, if all clusters contain the same number of elements and the worst result will be if all clusters contain just one element with the exception of one cluster which contains the remaining elements. SV, the sum of variances, measures the homogeneity of the clusters, which considers intra-cluster distances. Thus, it assesses low variance clusters which are compact (↦ Requirement 2) and is defined according to literature (e.g., (Faber, 1994)). A low value is therefore better than a higher one. MSC, the mean silhouette coefficient, considers compact (cf. SV) and clearly separated clusters (↦ Requirements 2 and 3). The possible results range from − 1 to 1 where 1 signals the optimum and thus clusters are built well apart from each other and are clearly distinguished. 0 shows that clusters are indifferent and − 1 is the worst result, where clusters are wrong assigned (e.g., many overlapping cluster ranges).

## Assessment Based on Quality Metrics

### Implementation of Algorithms

In order to evaluate the quality of Algorithm 1 (DDCAL), we compare it to a set of existing algorithms such as kmeans++ based on the quality metrics defined in Section 2.2. Furthermore, the parameters of DDCAL are evaluated.

In Section 3.2, the comparison is based on artificial data sets with different distributions and on these distributions, the evaluation of the DDCAL parameters is performed in Section 3.3. Finally, Section 3.4 compares the algorithms on real-world data sets. For this, we implemented Algorithm 1.^4^ as well as a selection of existing algorithms for comparison.

We used Python 3.9 for implementing all algorithms. For data operations, the numpy framework (version 1.21.2) was used. Because every algorithm produces different outputs, like clustered data points, centroids, breaks, or extrema on a curve, we converted each output to a uniform list of resulting elements, where each element contains a cluster number and a data point. Through these conversion steps, the performance of an algorithm may differ and a comparison of runtime should be considered with caution. Nevertheless, long runtimes when comparing different algorithms on huge data sets are pointed out in this work. Further details on the implementation of all algorithms can be found in Appendix B.

### Evaluation Based on Artificial Data with Different Distributions

We first want to understand how the results produced by Algorithm 1 relate to the distribution of the underlying data, also in comparison with k-means++, Jenks natural breaks, head/tail breaks, DBSCAN, KDE, GMM, mean shift, largest gaps, Gaussian kernel k-means, k-medoids, and trimmed k-means.

We generate artificial data sets of 1000 data points for different distributions. One thousand data points seem to result in meaningful data distributions to be distinguished from each other. Each data set contains at least one-third non-unique data points (cf. Section 1) to represent roughly a real-world data set. Moreover, when clustering the data sets by using *M* = 10 targeted clusters, each cluster contains about 100 data points if the results are evenly distributed over the clusters. Therefore, built clusters are small enough to be still be observed manually but are big enough to represent meaningful results. For representing different distributions, we have chosen well-known statistical distributions, to cover a broad range of possible data sets and to show how each algorithm, especially DDCAL, performs differently on different distributions. The following data sets are also accessible with details at^5^ normal distribution with 369 unique data points, gumbel distribution with 393 unique data points, uniform distribution with 558 unique data points, exponential distribution with 587 unique data points, and two peaks distribution with 362 unique data points which consists for two randomly generated normal distributions where the first has 300 and the second has 700 data points (cf. Table 1).

**Table 1:** Algorithm results for SED if NUC= 1 from different artificially generated distributions

Algorithm	Normal	Gumbel	Uniform	Exponential	Two Peaks
					
DDCAL	**6.69e + 19**	**7.08e + 19**	**9.93e + 19**	**5.29e + 19**	3.97e + 19
k-means++	8.50e + 18	1.16e + 19	9.47e + 19	1.29e + 18	1.61e + 19
variance of 10 trials	2.29e + 36	1.83e + 37	2.79e + 36	1.49e + 34	3.90e + 36
Jenks natural breaks	6.24e + 18	2.56e + 18	9.37e + 19	1.63e + 18	1.84e + 19
head/tail breaks	−	−	−	−	−
DBSCAN	7.08e + 04	2.37e + 04	−	−	3.86e + 08
KDE	1.48e + 14	7.89e + 11	5.73e + 19	4.96e + 10	3.23e + 14
GMM	8.33e + 18	9.37e + 17	7.43e + 19	1.30e + 18	1.78e + 19
variance of 10 trials	1.42e + 37	8.86e + 36	3.04e + 37	3.90e + 34	4.94e + 37
mean shift	−	−	−	5.64e + 14	−
largest gaps	2.35e + 05	7.90e + 03	3.25e + 06	3.51e + 05	4.74e + 08
Gauss. kernel k-means	2.69e + 19	1.45e + 19	8.83e + 19	4.35e + 19	**5.87e + 19**
variance of 10 trials	1.25e + 37	5.72e + 35	4.37e + 35	7.70e + 38	1.36e + 38
k-medoids	1.43e + 19	1.99e + 18	8.31e + 19	1.61e + 18	2.92e + 19
variance of 10 trials	2.00e + 38	6.73e + 37	8.89e + 36	1.05e + 36	1.12e + 38
trimmed k-means O-	2.69e + 19	2.06e + 19	3.36e + 19	1.37e + 19	1.90e + 19
variance of 10 trials	8.16e + 34	1.19e + 35	1.05e + 35	9.06e + 34	4.01e + 34
trimmed k-means O+		6.42e + 19	9.73e + 19	2.97e + 19	
variance of 10 trials	4.14e + 35	1.29e + 36	3.55e + 34	5.31e + 35	2.21e + 35

Number of elements for each distribution = 1000 and *M* = 10 where max SED = 1.00e + 20 (except for trimmed k-means O- (where O- means without outliers), which means that a defined percentage (= 10%) of elements to cluster were filtered because they were considered as outliers). Bold values indicate the best results if NUC= 1.0

We compare existing clustering algorithms with DDCAL by using *M* = 10 on the artificial data sets with different distributions. The results are shown in Tables 1, 2 and 3 by comparing based on metrics SED, SV, and MSC (cf. Section 2.2, and more details of the results can be found here.)^6^ The parameters set for the different algorithms for distributions to produce these results are shown in Table 4. The default parameters, which are not changed regarding a particular distribution, except for DDCAL which is described in the following, are shown in Appendix 2, for each particular algorithm. Also for stochastic algorithms, where the results depend on trial because of random methods in the particular algorithm, the variances from 10 trials are shown. The results show one execution. k-medoids and Gaussian kernel k-means result in the highest variances and thus the worst results. Furthermore, for k-medoids, metric SED showed the highest variance on all distributions, with exception of the exponential distribution, where Gaussian kernel k-means has the highest variance. The trimmed k-means algorithms lead to the lowest variances, with the exception of metric SV on uniform distribution with trimmed k-means O+. Thus, the trimmed k-means algorithms perform best and also k-means++ shows low variances over all data sets.

**Table 2:** Algorithm results for SV, if NUC = 1, from different artificially generated distributions

Algorithm	Normal	Gumbel	Uniform	Exponential	Two peaks
DDCAL	3.87e–01	7.88e–01	5.51e–01	1.53e + 01	3.69e–01
k-means++	5.25e–01	8.12e–01	5.12e–01	7.75e + 00	**2.49e–01**
Variance of 10 trials	3.04e–05	5.77e–04	2.11e-05	2.90e–01	3.36e–06
Jenks natural breaks	5.22e–01	8.11e–01	**5.07e–01**	9.57e + 00	6.68e-01
Head/tail breaks	−	−	−	−	−
DBSCAN	8.59e–01	1.17e + 00	−	−	4.54e–01
KDE	**3.15e-01**	**5.17e–01**	5.40e–01	5.21e + 00	2.75e–01
GMM	5.26e–01	7.32e–01	5.46e–01	6.88e + 00	2.50e–01
Variance of 10 trials	5.68e–03	6.23e–04	1.98e–04	7.15e–01	2.38e–05
Mean shift	−	−	−	**4.57e + 00**	−
Largest gaps	1.21e + 00	1.42e + 00	3.39e + 00	1.03e + 01	4.68e-01
Gaussian kernel k-means	4.66e–01	8.13e–01	5.35e–01	1.47e + 01	2.84e–01
Variance of 10 trials	9.98e–05	8.31e–08	2.32e–07	5.23e + 01	1.99e + 00
k-medoids	5.32e–01	8.02e–01	5.23e–01	7.85e + 00	2.55e–01
Variance of 10 trials	6.07e–03	9.23e–04	9.99e–05	4.04e + 00	1.46e–05
Trimmed k-means O–					
Variance of 10 trials	2.44e–08	4.89e–08	9.85e–07	7.12e-06	4.06e-08
Trimmed k-means O+	4.22e + 00	4.89e + 00	9.33e + 00	1.72e + 01	4.14e + 00
Variance of 10 trials	6.66e–05	6.06e–08	1.02e–02	4.64e–06	1.11e–04

Number of elements for each distribution = 1000 and *M* = 10. Bold values indicate the best results if NUC= 1.0

**Table 3:** Algorithm results for MSC if NUC= 1, from different artificially generated distributions

Algorithm	Normal	Gumbel	Exponential	Two peaks	
DDCAL	0.5	0.52	0.53	0.51	0.5
k-means++	**0.53**	0.53	**0.56**	0.57	**0.55**
Variance of 10 trials	5.05e-07	2.45e-06	1.36e-05	1.18e-06	1.20e-06
Jenks natural breaks	**0.53**	0.53	**0.56**	0.57	**0.55**
Head/tail breaks	−	−	−	−	−
DBSCAN	0.25	0.28	−	−	0.35
KDE	0.44	0.46	0.53	0.56	0.43
GMM	**0.53**	0.53	0.52	0.57	**0.55**
Variance of 10 trials	1.93e-05	7.06e-06	1.13e-04	1.01e-04	4.21e-05
Mean shift	−	−	−	0.54	−
Largest gaps	0.26	**0.56**	0.17	**0.62**	0.36
Gaussian kernel k-means	**0.52**	**0.53**	**0.54**	**0.49**	**0.52**
Variance of 10 trials	1.48e-05	3.98e-06	1.32e-08	2.62e-03	3.76e-04
k-medoids	**0.52**	**0.52**	**0.55**	**0.57**	**0.52**
Variance of 10 trials	2.39e-05	6.79e-05	3.53e-05	4.97e-05	1.36e-04
Trimmed k-means O–				**0.56**	
Variance of 10 trials	1.09e-07	1.39e-08	7.87e-07	1.11e-06	1.99e-08
Trimmed k-means O+	**0.42**	**0.44**	**0.46**	**0.51**	**0.44**
Variance of 10 trials	1.84e-07	2.12e-07	2.42e-08	2.50e-08	2.42e-08

Number of elements for each distribution = 1000 and *M* = 10. Bold values indicate the best results if NUC= 1.0

**Table 4:** Algorithm parameters which were set for the different distributions as shown in Tables 1, 2, and 3

Algorithm	Normal	Gumbel	Uniform	Exponential	Two peaks
DDCAL					
q_tolerance=	0.45	0.45	0.1	0.1	0.45
DBSCAN					
min_pts=	1,	1,	1,	1,	1,
eps=	0.09	0.11	0.04	0.47	0.07
KDE					
h=	0.07	0.08	0.14	0.30	0.02
Mean shift					
q=	0.002	0.002	0.002	0.214	0.005
Gauss. kernel k-means					
var=	13.39	15.81	26.38	0.40	0.31
Trimmed k-means O-/+					
trim=	0.1	0.1	0.1	0.1	0.1

For DDCAL (cf. Algorithm 1), we use the following input parameter values, i.e., boundary_min= 0.1, boundary_max= 0.49, num_simulation= 20, and q_tolerance_increase_step= 0.5. The parameter q_tolerance is set based on the distribution: for normal, gumbel, and two peaks distribution q_tolerance= 0.45 and for uniform and exponential with q_tolerance= 0.1. The setting of the input parameters is discussed in Section 3.3.

For other algorithms which need input parameters, we implemented simulation methods which aim to maximize the result of the metric SED if the precondition of NUC= 1 is fulfilled. Further details on each algorithm are described in Appendix 2.

The algorithm containing the best result is highlighted by a bold value in each table and on each distribution. We omit results and consequently algorithms which produce less than 10 clusters (i.e., NUC< 1) due to a fair comparison of the different algorithms. Also, results from trimmed k-means are excluded in the evaluation of the results because the algorithm filters outliers. The parameter of the trim factor is set to 0.1 which means that for 10% of the data to cluster, a separate cluster is built which contains identified outliers of the data set. This behavior violates 2 of 10 requirements set out in Section 1: (6) to avoid filtering/omitting of elements by the cluster algorithm and (5) to avoid overlapping cluster ranges. The latter is violated, because outliers are thrown in a separate cluster which contains likely elements from all areas of the data set. For this reason, bold values from trimmed k-means are highlighted in color blue if the result is best among the compared algorithms. We executed trimmed k-means twice on each data set, where the postfix O- indicates that only results without outliers are considered for metrics SED, SV, and MSC in order to avoid results with overlapping clusters as described before. Therefore, if 10% of the data points in the data set are trimmed, only the remaining 90% of the data points with their built clusters are evaluated. Hence, when *M* = 10 clusters are targeted, as described before, the actual targeted number on the algorithm is set to *M*+ 1 (i.e., 11). From the actually built clusters, only *m*-1 clusters, without the 1 outlier cluster are considered with the evaluation metrics. With this approach, the trimming of outlier data to cluster is performed, but no overlapping cluster ranges are produced. In contrast, if the postfix O+ is shown for trimmed k-means, also the cluster which contained the filtered outliers is considered based on metrics SED, SV, and MSC. However, using this approach, the appearance of overlapping cluster ranges is likely and thus it might not be useful for certain use cases (cf. Data Set 1 in Appendix 1).

In Table 1, the SED results are shown where the highest values represent the best ones. DDCAL performs best on the normal, gumbel, uniform, and exponential distribution and is slightly behind Gaussian kernel k-means (average rank of 2.2) on the two peaks distribution, resulting in an average rank of 1.2. trimmed k-means O+ performs also good on all distributions and best on normal and two peaks distribution. However, as mentioned before, overlapping clusters are produced on all distributions due to the outlier cluster, which contained 10% of the whole data set to cluster.

Table 2 shows the result for SV, where the lowest results indicate the best performance of an algorithm. DDCAL performs below average, except on the normal (rank 2) and gumbel (rank 3) distribution. In comparison, when calculating the average rank of all distributions, KDE performed best with 2.4, followed by k-means++ with 3.4 and GMM with 3.6. DDCAL had an average of 5.2 and was ranked as “6th” best algorithm, out of 8.

Table 3 shows the results of metric MSC, where the highest value shows the best result. k-means++, Jenks natural breaks, and GMM perform best on all distributions. These algorithms achieve always the first or second place, with the exception of GMM on the uniform distribution with only rank 7. Note that equal results have the same rank. DDCAL ranks 5 on uniform distribution, 6 on normal, gumbel, and two peaks distribution and 7 on the exponential distribution. By calculating the average rank of all distributions, k-means++ and Jenks natural breaks share the first place as best algorithms with 1.4, followed by GMM with 2.6 and k-medoids with 4. DDCAL scores below the average with an average rank of 6. Only KDE achieves a lower result with 6.6. MSC ranges from − 1 to + 1 and in most cases, except for the exponential distribution, the results for DDCAL differ from the results of the other algorithms on the second decimal place. Hence, we can still say that DDCAL performs close to the best performing algorithms.

When putting the ranks of all metrics on each algorithm together, by calculating the average rank on each distribution, DDCAL and Gaussian kernel k-means perform best on the normal distribution with an average order value of 3.0 (e.g., for DDCAL: $\frac {1+2+6}{3}$) from SED, SV, and MSC. On the gumbel distribution, DDCAL and GMM perform best with 3.3. For the other distributions, DDCAL performs worse than on the normal and gumbel distribution, where, for example, on the uniform distribution, k-means++ and Jenks natural breaks have the best average rank with 1.7 and DDCAL is on “5th” place. When looking at the exponential distribution, GMM scores best with 3.0 and DDCAL performs poorly with the “7th” place and an average rank of 5.3. Finally, on the two peaks distribution, k-means++ and GMM perform best with 2.7. DDCAL ranks 6 with an average score of 4.7.

Additionally to the ranking techniques as shown above and to get an even better picture of the effectiveness of the algorithms, we consider the differences between the scores for metrics SED, SV, and MSC by each algorithm. First, we normalize the results for each of the three metrics for each distribution by using the feature scaling method min-max normalization with the equation from Section 2 to obtain scores, ranging between 0 and 1, where 1 shows the best result, e.g., on SED for normal distribution DDCAL= 1.0 (indicates the best rank), KDE = 0.59 (indicates that the result is about less the half than the best ranking result), and largest gaps = 0 (indicates worst rank). On SV, we have to do an extra step after normalization, because the best result has the lowest score. Thus, we subtract each normalized result from 1, e.g., for KDE: 1 − 0 = 1. For metrics SED on the normal distribution, we discover that, for example, DDCAL has a normalized result of 1 and the second best is Gaussian kernel k-means with only 0.40. Nearly the same picture is shown when we look at the gumbel distribution, where the DDCAL has a normalized result of 1 and the second is Gaussian kernel k-means with 0.20. With the exception of the two peaks distribution, where DDCAL ranks second best 0.68 behind Gaussian kernel k-means, DDCAL performs best on all tested distributions. When DDCAL is compared based on metric SV, the results are about average, i.e., 0.91 on normal distribution, 0.69 on gumbel distribution, 0.98 on uniform distribution, and 0.71 on two peaks distribution, except for the exponential distribution with a result of 0. The normalized results for MSC show nearly the same picture as shown for the previous metric SV: 0.89 on normal distribution, 0.6 on gumbel distribution, 0.92 on uniform distribution, and 0.74 on two peaks distribution. Similarly to SV, DDCAL shows an below-average performance with with 0.15 for MSC with the exponential distribution. By averaging the normalized results from all metrics, where each metric is considered with equal weight, on each distribution, DDCAL performs best on normal distribution with 0.94 ($\frac {1+0.92+0.89}{3}$), followed by Gaussian kernel k-means with 0.73. On gumbel distribution, DDCAL performs best with an average of 0.77, followed by Gaussian kernel k-means with 0.53 which was close to k-means++ with 0.51. For the uniform distribution, the first three algorithms lead to similar results, i.e., 0.984 for k-means++, 0.981 for Jenks natural breaks, and 0.97 for DDCAL. On the exponential distribution, KDE performs best with an average of 0.51 and DDCAL ranks 7 with a score of 0.38. Finally, on the two peaks distribution, Gaussian kernel k-means has the highest average score with 0.92 and DDCAL ranks 4th place with a score of 0.71. Based on the sum of the normalized results over all distributions from all metrics the algorithms can be ranked as follows: DDCAL with 3.77 (0.94 + 0.77 + 0.97 + 0.38 + 0.71), Gaussian kernel k-means with 3.41, k-means++ with 3.35, GMM with 3.26, k-medoids with 3.25, Jenks natural breaks with 2.91, KDE with 2.67, and largest gaps with 0.99.

Through the analysis of the different metrics, it seems that DDCAL performs well on normal and gumbel distributions. By considering the mechanics of DDCAL, outliers above or below a threshold are the first generated clusters. After every iteration, these outliers are removed continuously from the input list and assigned to clusters. Through this procedure, the input list becomes more evenly distributed. DDCAL builds only a cluster in an iteration if on both sides, above and below a threshold, outliers are identified as potential clusters, containing a minimum amount of data points. Hence, the algorithm performs better, if just one peak with two tails exists in the distribution of a given data set, and also because outliers are considered as local in DDCAL instead of global. DDCAL can be improved on data sets with two or more peaks by a pre-processing step which cuts the data set into two or more slices where every data set contains one distribution.

### Evaluation of DDCAL Parameters on Different Distributions

**Fig. 3:**
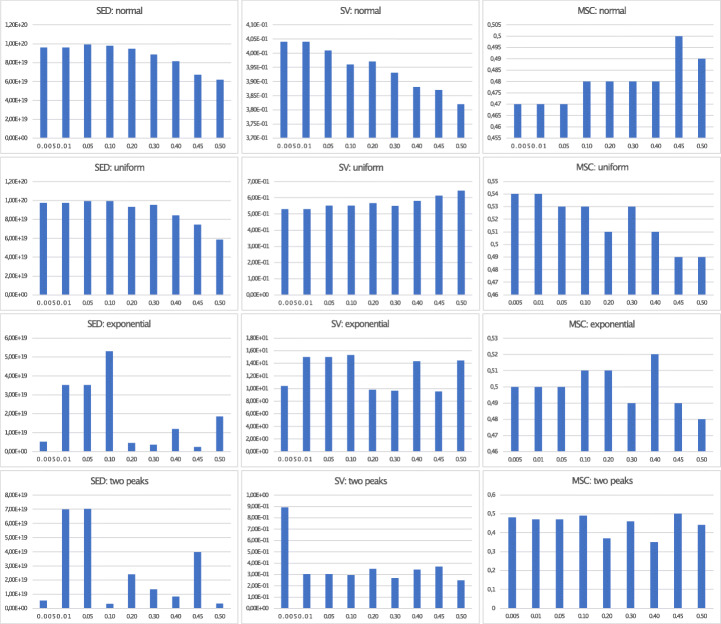
Visualization of metrics SED, SV, and MSC for DDCAL on different distributions with different q_tolerance parameters and *M* = 10

**Fig. 4:**
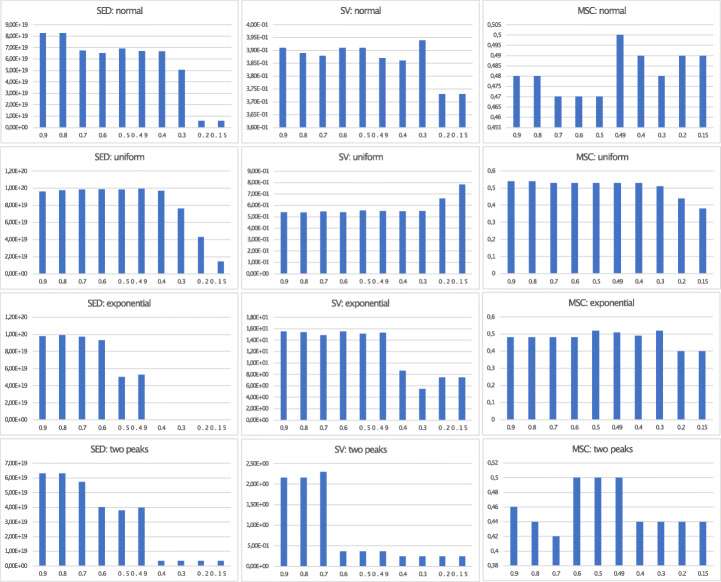
Visualization of metrics SED, SV, and MSC for DDCAL on different distributions with different boundary_max parameters and *M* = 10

**Fig. 5:**
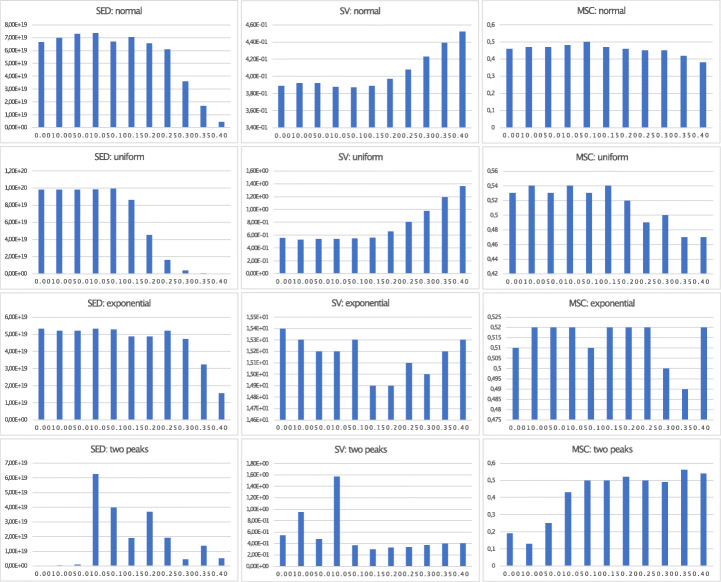
Visualization of metrics SED, SV, and MSC or DDCAL Advanced on different distributions with different boundary_min parameters and *M* = 10

In the previous sections, we proposed particular values for parameters on DDCAL which we also used for evaluation. These default parameters (boundary_min= 0.1, boundary_max= 0.49, num_simulation= 20, q_tolerance_increase_step= 0.5, q_tolerance= 0.45 resp. 0.1) are chosen because of the results from simulations on different distributions, as shown in Figs. 3, 4, and 5 for the DDCAL algorithm (more details of the results can be found here.)^7^ We left out the gumbel distribution because the impact on the results, by setting different input parameters, is nearly the same as on the normal distribution.

At first, we evaluate different settings for parameter q_tolerance, which defines the minimum quantity of elements for building a cluster. As shown in Fig. 3, we tested different values between 0.005 and 0.5 with the observation that DDCAL performs well on normal (also gumbel) and two peaks distribution, when the parameter is set to 0.45. On the uniform and exponential distribution, 0.1 performs well. On the two peaks and exponential distribution, there exist many jumps in the results of the metrics. Therefore, a high q_tolerance factor of, for example, 0.4 on the exponential distribution can perform well, resulting in a lower SED value and the best value for MSC.

Overall, we recommend for real-world data sets, which are similar to the tested distributions, to set q_tolerance to a higher value like 0.45 on normal and gumbel distribution and to a lower value, like 0.1 on a uniform distribution. On exponential and two peaks distribution, we recommend to test both high and low q_tolerance factors, such as 0.45 and 0.1.

The q_tolerance has huge impact on the results on different distributions and can be used to adjust the trade-off between the results of SED and the classical clustering metrics SV and MSC in small steps, e.g., by setting the parameter from 0.45 to 0.5 on the normal distribution, where a higher q_tolerance factor decreases SED (which is still high) but produces better results for SV (which gets lower) and MSC (which increases).

For further tests on different parameters, we use the best performing q_tolerance setting on each distribution as discovered before. Note, we also performed tests on the uniform and exponential distribution by setting q_tolerance to 0.45, even though our observation showed that a lower q_tolerance factor seems to perform better. In most cases, except for testing the parameter boundary_min on exponential distribution, similar curves in the diagram are produced and the results on all three metrics decrease.

For DDCAL, we consider boundaries up to 0.49 as meaningful with respect to achieving fair results and because clustering outliers above 49%, these results cannot be considered as “outliers,” as they make up more than half of the remaining data points on each iteration step. The results depicted in Fig. 4, for which different boundary_max parameters (ranging from 0.15 to 0.9) are tested, show that the overall performance is good with a maximum boundary of 0.49. Thus, we recommend to use the parameter boundary_max= 0.49 on all distributions. The exponential distribution with a higher value performs also good and even better on SED.

When investigating the parameter boundary_min, where the results are shown in Fig. 5, it turns out that to start with a 10% boundary (minimum boundary) for clustering outliers, seems to be a good balance between a high SED value and good results on SV, and MSC. Starting with 15%, leads to DDCAL performing even better in some cases. For example, on the exponential distribution boundary_min= 0.15 yields better results than = 0.1 on SV and MSC on slight costs of SED, but on normal distribution this observation is reversed, where SED is slightly higher on costs of SV and MSC. Therefore, we recommend to use boundary_min= 0.1 on all distributions and to test boundary_min= 0.15 for fine tuning.

With the parameter num_simulations, as described in Algorithm 1, we defined the number of simulation steps for testing different boundaries on each iteration in DDCAL. These boundaries are evenly spaced values from the defined input parameters boundary_min to boundary_max with the defined number of simulation steps, where we consider that 20 elements are sufficient to test all necessary boundaries.

This number is confirmed through testing different numbers of simulation steps (above and below) which show that less than 20 simulation steps decrease the results from the metrics and more than 20 simulation steps do not show significant improvements, but increase the runtime of the algorithm.

Also, the parameter q_tolerance_increase_step is tested with different values. A value of 0.5 seems to perform best, but slight changes have hardly any impact on the results. When setting q_tolerance_increase_step too low, more iteration steps are performed, which increases the runtime of the algorithm. A too high value such as 3 decreases the performance of the metrics, especially for SED, because clusters are built too early when the minimum quantity for building a cluster is reached immediately.

We tested DDCAL by changing more than one parameter at the same time, but the results from the metrics always decrease. Thus, we recommend to change only one parameter value and to use the recommendations as described before. In detail, parameter q_tolerance can be used as pivot for better results. For slight improvements, the parameter boundary_min can be changed, as well. An exception, where the boundary_min changes the results completely, is shown on the two peaks distribution by setting boundary_min to the value 0.35 instead of 0.1, which shows, when comparing DDCAL with 7 other algorithms (cf. Tables 1, 2, and 3) the following results: the performance of SED declines from ranks 2 to 6. The rank of SV remains 6 and MSC improves from 6 to the best performing algorithm (rank 1) with an MSC value of 0.56.

**Discussion:** Throughout the paper, we assume *M* = 10, but other *M* values were tested on the data sets described in Section 3.2. On data set normal distribution, for example, we compared DDCAL with Jenks natural breaks on different values of *M* (more details of the results can be found here.)^8^ We have chosen Jenks natural breaks for comparison, because it was the best performing algorithm in Section 3.2 (cf. Tables 1, 2, and 3) with stable results on *M* = 10. Jenks natural breaks ranks third behind DDCAL and k-means++ when calculating the average rank for each distribution (cf. Section 3.2) and ranks sixth behind DDCAL and the stochastic algorithms Gaussian kernel k-means, k-means++, GMM, and k-medoids when calculating the normalized results.

For DDCAL, we used the default parameters (boundary_min= 0.1, boundary_max= 0.49, num_simulation= 20, q_tolerance_increase_step= 0.5, q_tolerance= 0.45) as stated above. First we observe that for *M* ∈ {3,4}, Jenks natural breaks shows better results for SED, SV, and MSC. For *M*∈ {5, 6, 8, 12, 15, 30}, SED and SV perform better for DDCAL, but MSC turns out slightly worse than Jenks natural breaks, which shows the same observation as with *M* = 10 (cf. Tables 1, 2, and 3). As shown before, in some cases, MSC can be improved without costs of other parameter performance, when setting the parameter boundary_min to = 0.15 instead of 0.1. For *M* = 12, for example, SED improves from 7.78e + 22 to 9.01e + 22, SV remains the same value of 3.69e-01, and MSC improves from 0.48 to 0.5 where Jenks natural breaks has values of SED= 8.11e + 21, SV= 4.90e-01 and MSC= 0.54. The same observation of the improvement of DDCAL by changing this parameter is discovered for *M* = 6, where all metrics improve. By changing the parameter for other values of *M*, the performance of the metrics decreases.

When setting *M* to a high value of, e.g., 100 (with just 1000 data points in the data set), Jenks natural breaks produces 100 clusters where DDCAL produced only 44 clusters. The lower number of produced clusters in the output results from the termination of the algorithm, if no data is left for clustering (cf. Algorithm 1 step #36) and hence it makes no sense to produce more clusters. Because of the fewer clusters, the results of DDCAL rank behind Jenks natural breaks for metrics SED (1.51e + 52 vs. 3.50e + 90) and SV (3.97e-01 vs. 3.86e-01) and better for metric MSC (0.53 vs. 0.5). However, a fair comparison can only be made if both algorithms produce the same number of clusters. When setting the parameter boundary_min to 0.01 instead of 0.1, DDCAL produces 100 clusters, where all metrics perform better than Jenks natural breaks (SED= 1.17e + 97, SV= 3.40e-01, and MSC= 0.54). By lowering boundary_min, it is more likely that the envisaged number of data points per cluster is found, because even low deviations of the data points are counted as outliers (cf. Algorithm 1 step #7). In other words, if not a sufficient number of clusters (*m*<*M*) are built after executing DDCAL, boundary_min can be lowered, to fill the target number of clusters (*M*) with data points.

### Evaluation Based on Real-world Data Sets

To compare DDCAL with existing algorithms as described in Sections 3.1 and 3.2, we use four real-world data sets from different domains in order to show the wide range of possible applications, i.e., Data Set 1 on search processes, Data Set 2 on US population, Data Set 3 on star distances, and Data Set 4 on weather (all data sets are described in Appendix 1).

The results of comparing selected algorithms (cf. Appendix B) are shown in Tables 5, 6, 7, and 8 (more details of the results can be found here).^9^ Like shown on Tables 1, 2, and 3 (cf. Section 3.2), each table contains variances from 10 trials for stochastic algorithms, where the yielded results depend on the trial because of random methods in the particular algorithm. The results of each of these algorithms represent one execution for the actual comparison among the other algorithms.

**Table 5:** Results for Data Set 1 (process mining) by using *M* = 10 where max SED = 1.40e + 05 (except for trimmed k-means O- (where O- stands for without outliers), which means that a defined percentage (= 10%) of elements to cluster were filtered because they were considered as outliers)

Algorithm name	NUC (max)	SED (max)	SV (min)	MSC (max)
DDCAL q_tol= 0.45 boundary_min= 0.1	**1.0**	**4.08e + 04**	1.68e + 02	**0.78**
k-means++	**1.0**	9.60e + 02	**5.81e + 00**	0.7
Variance of 10 trials	0	0	0	0
Jenks natural breaks	**1.0**	2.18e + 03	1.96e + 06	0.35
Head/tail breaks	0.2	3.20e + 01	4.31e + 02	0.96
DBSCAN min_pts= 1 eps= 3	**1.0**	9.60e + 02	**5.81e + 00**	0.7
KDE h = 0.1	0.2	3.20e + 01	4.31e + 02	0.96
GMM	**1.0**	3.51e + 03	1.41e + 01	0.59
Variance of 10 trials	0	3.91e + 06	1.09e + 01	1.26e-04
Mean shift q = 0.1	0.2	3.20e + 01	4.31e + 02	0.96
Largest gaps	**1.0**	9.60e + 02	1.96e + 06	0.63
Gaussian kernel k-means var= 20.39	**1.0**	6.32e + 03	2.38e + 0	0.61
Variance of 10 trials	0	1.18e + 08	1 2.40e + 03	1.08e-02
k-medoids	**1.0**	3.51e + 03	1.41e + 01	0.59
Variance of 10 trials	0	5.31e + 04	5.80e + 00	1.43e-04
Trimmed k-means O- trim= 0.1	**1.0**	2.27e + 03		0.69
Variance of 10 trials	0	0	1.18e-32	4.44e-33
Trimmed k-means O+ trim= 0.1	**1.0**	1.36e + 04	1.77e + 06	0.6
Variance of 10 trials	0	0	6.62e + 11	7.84e-05

Bold values indicate the best results if NUC= 1.0

**Table 6:** Results for Data Set 2 (U.S. population 2018) by using *M* = 10 where max SED = 6.25e + 06 (except for trimmed k-means O- (where O- stands for without outliers), which means that a defined percentage (= 10%) of elements to cluster were filtered because they were considered as outliers)

Algorithm name	NUC (max)	SED (max)	SV (min)	MSC (max)
DDCAL q_tol= 0.45 boundary_min= 0.15	**1.0**	**3.70e + 06**	6.69e + 13	0.49
k-means++	**1.0**	2.02e + 05	1.96e + 12	**0.61**
Variance of 10 trials	0	5.28e + 06	7.46e + 18	2.53e-06
Jenks natural breaks	**1.0**	3.81e + 05	4.31e + 13	0.4
Head/tail breaks	0.4	1.45e + 03	3.82e + 13	0.56
DBSCAN min_pts= 1 eps= 565973.5	**1.0**	3.59e + 03	2.67e + 12	0.42
KDE h = 428947	**1.0**	5.24e + 04	1.84e + 12	0.52
GMM	**1.0**	3.14e + 05	2.50e + 12	0.56
Variance of 10 trials	0	7.96e + 09	9.85e + 22	9.79e-04
Mean shift q = 0.074	**1.0**	8.74e + 04	**1.79e + 12**	0.55
Largest gaps	**1.0**	3.18e + 04	3.11e + 13	0.51
Gaussian kernel k-means var= 559009.01	**1.0**	3.63e + 06	1.21e + 14	0.41
Variance of 10 trials	0	7.90e + 11	1.24e + 28	9.21e-03
k-medoids	**1.0**	4.03e + 05	2.38e + 12	0.56
Variance of 10 trials	0	2.12e + 10	9.63e + 22	4.35e-04
Trimmed k-means O- trim= 0.1	**1.0**	4.35e + 05		
Variance of 10 trials	0	1.11e + 09	1.36e + 20	3.50e-05
Trimmed k-means O+ trim= 0.1	**1.0**	3.05e + 06	6.29e + 13	0.6
Variance of 10 trials	0	3.98e + 10	1.36e + 20	2.44e-05

Bold values indicate the best results if NUC= 1.0

**Table 7:** Results for Data Set 3 (distances to stars) by using *M* = 10 where max SED = 6.00e + 40 (except for trimmed k-means O- (where O- stands for without outliers), which means that a defined percentage (= 10%) of elements to cluster were filtered because they were considered as outliers)

Algorithm name	NUC (max)	SED (max)	SV (min)	MSC (max)
DDCAL q_tol= 0.45 boundary_min= 0.1	**1.0**	**3.22e + 40**	1.59e + 04	0.55
k-means++	**1.0**	5.71e + 39	**1.04e + 04**	0.57
Variance of 10 trials	0	3.13e + 78	1.49e + 05	6.99e-06
Jenks natural breaks	**1.0**	6.08e + 39	8.58e + 07	0.57
Head/tail breaks	0.2	1.12e + 09	3.63e + 04	1.0
DBSCAN min_pts= 1 eps= 8.34	**1.0**	4.25e + 24	3.32e + 04	**0.69**
KDE h = 0.1	0.2	1.12e + 09	3.63e + 04	1.0
GMM	**1.0**	8.92e + 39	1.06e + 04	0.57
Variance of 10 trials	0	1.17e + 79	4.51e + 05	2.26e-05
Mean shift q = 0.068	**1.0**	2.24e + 34	1.40e + 04	0.58
Largest gaps	**1.0**	4.66e + 24	8.58e + 07	0.68
Gaussian kernel k-means	−	−	−	−
k-medoids	−	−	−	−
Trimmed k-means O- trim= 0.1	**1.0**	1.29e + 40		0.58
Variance of 10 trials	0	5.58e + 76	2.38e + 01	−
Trimmed k-means O+ trim= 0.1	**1.0**		2.05e + 04	0.53
Variance of 10 trials	0	2.39e + 77	2.34e + 01	−

Bold values indicate the best results if NUC= 1.0

**Table 8:** Results for Data Set 4 (weather) — min temperatures by using *M* = 10 where max SED = 4.31e + 15 (except for trimmed k-means O- (where O- stands for without outliers), which means that a defined percentage (= 10%) of elements to cluster were filtered because they were considered as outliers)

Algorithm name	NUC (max)	SED (max)	SV (min)	MSC (max)
DDCAL q_tol= 0.1 boundary_min= 0.15	**1.0**	**3.91e + 15**	5.13e + 00	0.51
k-means++	**1.0**	3.19e + 15	4.82e + 00	0.55
Variance of 10 trials	0	5.07e + 28	3.09e-03	2.15e-05
Jenks natural breaks	**1.0**	3.52e + 15	4.84e + 00	0.54
Head/tail breaks	0.2	3.35e + 04	1.83e + 01	0.63
DBSCAN min_pts= 1 eps= 0.3	0.9	1.94e + 08	1.67e + 01	0.24
KDE h = 0.62	0.7	7.42e + 10	6.60e + 00	0.52
GMM	**1.0**	4.14e + 14	**4.48e + 00**	**0.56**
Variance of 10 trials	0	6.99e + 29	1.95e-02	1.77e-04
Mean shift q = 0.15	**1.0**	3.99e + 13	5.91e + 00	0.53
Largest gaps	**1.0**	5.15e + 09	1.67e + 01	0.19
Gaussian kernel k-means var= 1.61	**1.0**	3.40e + 15	4.75e + 00	**0.56**
Variance of 10 trials	0	0	1.97e-31	1.97e-33
k-medoids	**1.0**	4.39e + 14	**4.48e + 00**	**0.56**
Variance of 10 trials	0	7.63e + 29	1.86e-02	3.35e-05
Trimmed k-means O- trim= 0.1	**1.0**	1.38e + 15		
Variance of 10 trials	0	1.60e + 27	6.64e-05	1.60e-05
Trimmed k-means O+ trim= 0.1	**1.0**	3.59e + 15	7.72e + 01	0.46
Variance of 10 trials	0	1.29e + 28	6.08e + 01	5.21e-05

Bold values indicate the best results if NUC= 1.0

In the description of each table, the maximum SED value is listed for the data set to indicate how close each algorithm performs to this value. Bold values indicate the best results among the compared algorithms in a table if NUC= 1.0 and therefore *M* is reached (*m*=*M*). As described in Section 3.2, in the following, we discuss only those results with NUC= 1.0 and without trimmed k-means where bold values from this algorithm are highlighted in color blue if the result was best among the compared algorithms.

The default parameters for the comparison algorithms which are not changed regarding a particular data set are shown in Appendix 2. For the DDCAL algorithm, we used the default parameters (boundary_min= 0.1 resp. 0.15, boundary_max= 0.49, num_simulation= 20, q_tolerance_increase_step= 0.5, and q_tolerance= 0.45 resp. 0.1) as described in Sections 3.2 and 3.3. For Data Set 4, q_tolerance is set to 0.1 because of the uniform distribution and on all other data sets, q_tolerance is set to 0.45. For Data Set 2, boundary_min is set to 0.15 because the metrics SED, SV, and MSC perform better. For Data Set 4, boundary_min is set to 0.15, where SED performs a little weaker than 0.1, but the performance increases for metrics SV and MSC.

DDCAL achieves outstanding results given an equal distribution of the data points over the clusters (SED) for all real-world data sets, where DDCAL hit always rank 1 in comparison to all other algorithms. On SV and MSC, the performance of DDCAL is in the midfield or below.

On Data Set 1 (process mining), DCAL ranks 6 for SV 1 for MSC. k-means++ and DBSCAN yield the same output and thus the metrics have the same results. They both rank 1 for metric SV, 2 for MSC, and second last for metric SED which demonstrates a trade-off between SED and the classical clustering metrics SV and MSC. For the stochastic algorithms, k-means shows the same results for all trials and the variance for all metrics thus turns out as 0. Also, the trimmed k-means algorithms performs with no variance for SED. The Gaussian kernel k-means performs worst regarding variances from the 10 trials.

For Data Set 2 (U.S. population 2018), mean shift followed by KDE performed best on metric SV and DDCAL had the “9th” rank out of 10. Regarding metric MSC, DDCAL ranks on place number 7 and k-means++ ranks the first place, followed by GMM. Similar to the previous discussed data set, the variance of k-means++ is the lowest and thus the best and for Gaussian kernel k-means the variance is the highest and thus the worst on stochastic algorithms on this data set.

For Data Set 3 (Stars), with respect to metric SV, DDCAL ranks 4 out of 7 algorithms. k-means++ and GMM perform best. For MSC, DDCAL hits the last place where DBSCAN hits the first, and largest gaps the second place. When comparing the variances of the stochastic algorithms, the trimmed k-means algorithms perform best with low variances where GMM shows the highest variances after 10 trials.

Finally, on Data Set 4 (Weather), for metrics SV and MSC, DDCAL scores between the “6th” and the “7th” rank out of 8. GMM performs best for SV and MSC. k-medoids has the second best results for SV and the best ones for SV. Gaussian kernel k-means performs second best for MSC. For the stochastic algorithms, the variances of trimmed k-means O+ are the highest and thus worst among the compared algorithms. The variances for Gaussian kernel k-means are the lowest and thus the algorithm performs best when comparing the deviations on the metrics from 10 trials.

As for the synthetic data sets (cf. Section 3.2), we sum up all results for all metrics for each distribution and algorithm by using the feature scaling method min-max normalization using the equation provided in Section 2. The scores range from 0 to 1 where 1 indicates the best result. For Data Set 1 (process mining), DDCAL hit the first place, followed by k-means++ and DBSCAN. For Data Set 2 (U.S. population 2018), k-means++ performs best, followed by DDCAL and k-medoids. For Data Set 3 (Stars), DDCAL shows the best overall performance, followed by DBSCAN and GMM. Finally, for Data Set 4 (Weather), DDCAL performs best and is followed by k-means++ and Jenks natural breaks.

In summary, for metrics SV and MSC, none of the algorithms achieves a good ranking on all real-world data sets. In contrast, the SED results on DDCAL come close to the maximum SED value for each of the real-world data sets. Furthermore, the results show that there exists a trade-off between metric SED and the classical clustering metrics SV and MSC, as discussed earlier. Section 4 will illustrate why a high SED value from clustered results as pre-processing of 1d data for visualization is favorable based on different use cases, as also already mentioned in Section 1.

The comparison of the characteristics of the algorithms leads to the following observations: k-means++ shows a good overall performance on the real-world data sets. One reason is that the real-world data sets are similar to an exponential and uniform distribution, where the algorithm also achieves good results (cf. Section 3.2).

k-means++, GMM, Gaussian kernel k-means, k-medoids, and trimmed k-means (which was left out in the comparison) are struggling to be reproducible, because different results are yielded depending on the trial (Thrun, 2021). Thus, after each run, the clustered results differ and thus, do not produce stable results like DDCAL. This behavior produces a weak violation of one requirement set out in Section 1. We speak of a weak violation, because trial sensitive random methods can be set to a fixed number (e.g., the random_state parameter for k-means++ on the Python framework sklearn)^10^, with the effect that the random method will be deterministic and returns always the same results. However, this approach may not lead to the best results of an algorithm.

The variance of the before mentioned algorithms is shown for the metrics in Tables 1, 2, 3, 5, 6, 7, and 8 because we use random methods without restrictions. Head/tail breaks, DBSCAN, KDE, and mean shift do not entail any parameter to define the number of aimed clusters, *M*. Except for head/tail breaks, the aforementioned algorithms have input parameters for which we use simulation methods to maximize metric SED which implies to optimize *m*. We use the simulation method also in order to not exceed *M* for the particular algorithms. In terms of runtime, mean shift, trimmed k-means, and Gaussian kernel k-means are the slowest algorithms on the biggest Data Set 3. For 119,614 data points, the Gaussian kernel k-means is not able to terminate within 48 h, the trimmed k-means algorithms takes more than 15 h, and the mean shift algorithm was quite slow on this data set with an execution time of about 4 min without the simulation method. DDCAL has a runtime of about 3 s with its built-in simulation method (the algorithm has a complexity of O(n log n), by including the sorting step at the beginning. Regarding the observed runtime of the algorithms, it should be noted that their implementation in Python (cf. Section 3.1) may use different frameworks and data structures under the hood. This might cause different results in terms of performance. Thus, the observations regarding runtime should be considered with caution and interpreted as experimental run and are not further discussed. Also, k-medoids is not able to to produce results on the biggest Data Set 3, because the computer, where the algorithm was executed, ran out of memory.

## Application: Visualization of Data on Maps and Process Models

Getting the most insights out of the data can be supported by the “[t]ight integration of visual and automatic data analysis methods” (Keim et al., 2008); particularly, the model building can be seen as the iterative application of data mining methods and visual exploration.

Hence, we can identify pre-processing of 1d data for visualization on business process models and (cloropleth) maps as applications for DDCAL (Algorithm 1).

**Fig. 6:**
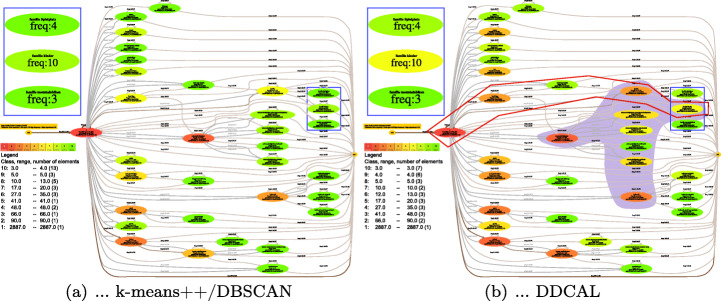
Process mining data set pre-processed by (a) k-means++/DBSCAN and (b) DDCAL

The visualization of business process models represented by directed graphs, as introduced in Section 1, can be enriched by data connected with the nodes of the graph. Data Set 1, for example, stores the number of search results for nodes in a process model that reflects the search behavior of customers. Figure 6 depicts the process model that is used as underlying structure for visualizing Data Set 1 using different clustering algorithms, i.e., k-means++ and DBSCAN in Fig. 6(a) and DDCAL in Fig. 6(b), for coloring each node based on their frequency, labeled as freq in each node. The results for all clustering algorithms are shown in Table 5, where k-means++ and DBSCAN had the same output. We have chosen k-means++ and DBSCAN to compare with the DDCAL because its overall normalized performance among algorithms from the metrics SED, SV, and MSC together was the second best performing with NUC= 1, where DDCAL performed best (cf. Section 3.4).

At first sight, both visualization results differ. In Fig. 6(a) which used k-means++ and DBSCAN for coloring the nodes, there is no difference between the most frequently occurred frequencies in the process model, namely between frequencies 3 and 4 which were assigned to the the same “green” color gradient. By contrast, in Fig. 6(b), there is a distinction between frequency 3 and 4 which is highlighted as a blue frame in the process model and for readability, and these nodes are magnified as well in a blue box on the left side. Such a distinction is important as the whole process model shows 33 nodes in total and nodes with frequency 3 appear 7 times (i.e., 21%) and nodes with frequency 4 appear 6 times (i.e., 18%). Furthermore, if a visualization tool for process models supports a filter option to filter out nodes with low frequencies, for example to show just frequencies > 4, the distinction between often occurred nodes with colors is important because an observer can see visually, which elements are affected from such a filtering step. Generally, a higher variety of colors apart from just “green” tones is shown in the process model which helps to recognize heat maps, for highlighting regions with a high frequency. Such an example is demonstrated through the violet highlighted region in the process model. This also enables a stronger distinction of execution frequencies and in the sequel execution behavior of users between these activities. Furthermore, a happy flow of process paths can be easily discovered at first glance through the coloring of nodes based on their frequencies in process models. A happy flow from process paths which contain more than 4 nodes is highlighted with a red frame.

Note that the clusters do not have to be necessarily mapped onto colors for visualization. For the process mining use case, a mapping onto edges in terms of stroke width is also conceivable.

**Fig. 7:**
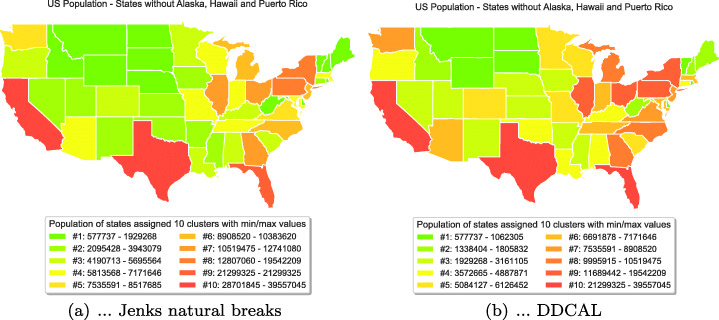
Process mining data set pre-processed by (a) Jenks natural breaks and (b) DDCAL

A different visualization is shown on Data Set 2. The goal on this data set is to visualize the data on a US map in order to distinguish the population sizes of different states. For visualization of a choropleth map, we downloaded the shape files of the US states from the year 2018 from census.gov.^11^ Then, the data is pre-processed by clustering using (a) Jenks natural breaks and (b) DDCAL. The results of all clustering algorithms were already introduced in Table 6 and discussed as well in Section 3.4. We used the algorithm Jenks natural breaks to compare with DDCAL because it was designed to visualize choropleth maps (Jenks, 1967). Figure 7 depicts the results for both algorithms. In Fig. 7(a), it is shown that the clustering by using Jenks natural breaks does not result in well distinguishable colors for states with low population sizes due to the “dominance” of a few states with high population size in the south and east. Thus, the majority of the states with lower populations (in green colors) can hardly be distinguished. Compared to Jenks natural breaks, the visualization in Fig. 7(b), by using the clustering algorithm DDCAL, the assigned colors to the states lead to a more fine-grained exploitation, enabling the distinction of the population sizes of more states, particularly of those with lower population sizes.

**Fig. 8:**
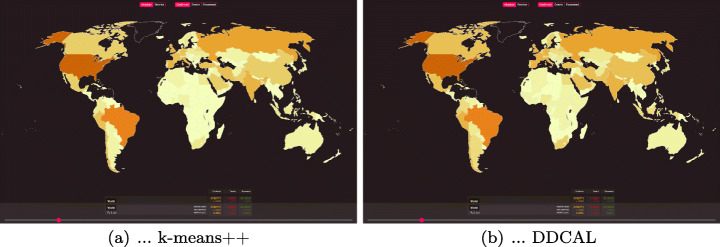
Corona visualization of absolute confirmed cases for the date 2020-06-05 from the Johns Hopkins CSSE data set pre-processed by (a) k-means++ and (b) DDCAL

Additionally, Data Set 5 features data on the Corona pandemic that has posed highest priority to monitor closely the infection development in order to enable quick reactions to (local) outbreaks and subsequent mitigation actions (Thomas et al., 2020). Several visualizations have been provided, for example, the dashboard of the World Health Organization (WHO).^12^ For the visualization with cluster pre-processing, we take the subset of absolute confirmed cases from 2020-06-05. That day, the US accounted for around 28% of all confirmed cases world-wide while the 52 African states captured by the data together account for around 2.6%. Hence, we can speak of outliers in the data. Figure 8 displays two visualizations of the data set that have been pre-processed by clustering and are displayed using the app.^13^ In (a), k-means++ is used for clustering, in (b) the DDCAL (cf. Section 2) algorithm to be proposed in this work. At first glance, it can be seen that in (a) there is almost no visual differentiation for African countries, whereas in (b) different clusters can be differentiated. For example, the cluster containing Egypt (0.5%) and South Africa (0.6%) can be distinguished from a cluster containing — among other countries — Nigeria (0.18%) and Algeria (0.15%) and furthermore, Mali (0.002%) can be distinguished from Niger (0.001%). The numbers in brackets denote the percentages of confirmed cases in the respective countries in relation to the number of confirmed cases world-wide.

## Related Work

The definition of clustering is still an open discussion (Estivill-Castro, 2002). In some ways, it is described to group similar data into a cluster for permitting a significant generalization (Bonner, 1964) and it helps in data mining to identify interesting structures in data (Thrun et al., 2020). A different clustering concept, which is not treated in this work, is conceptual clustering, which accepts a set of object descriptions to produce a classification scheme over observations (Fisher, 1987). Section 3 provides a detailed comparison of DDCAL with a set of related clustering algorithms, i.e., k-means++ (Arthur & Vassilvitskii, 2007) (Arthur & Vassilvitskii, 2006), Jenks natural breaks (Jenks, 1967), head/tail breaks (Jiang, 2013), DBSCAN (Ester et al., 1996), KDE (Scott, 2015), GMM (Reynolds, 2009), mean shift (Comaniciu & Meer, 2002), largest gaps, Gaussian kernel k-means (Dhillon et al., 2004), k-medoids (Park & Jun, 2009), and trimmed k-means (Cuesta-Albertos et al., 1997). Further algorithms which can be used for clustering of (1d) data points are, for example, hierarchical cluster methods like pairwise agglomerative clustering as described in Faber (1994). This method has a major drawback in terms of performance on large data sets because each iterative step requires multiple distance calculations. Many popular algorithms such as k-means have various modifications of their basic concept, e.g., the k-medoids algorithms (Thrun, 2018) which are designed to be less sensitive to outliers than the original algorithm. One implementation here is the PAM algorithm (Kaufman and Rousseeuw, 1990) which uses medoids instead of centroids, i.e., data points in the data set itself and the Minkowski distance. One-dimensional clustering can be used to generate choropleth maps (Wright, 1938) for visualizing how a measurement varies across a geographic area. Another use case for one-dimensional clustering is to generate heat maps, e.g., for visualizing the frequency of nodes or edges in directed graphs from mined processes by using process mining algorithms (van der Aalst, 2016), which are similar to choropleth maps, but with the difference that they do not use geographic areas. Fair clustering techniques such as Chierichetti et al. (2017) aim at minimizing distances between the data points within the clusters and maximizing the distances of the points between different clusters. On top of that, they try to respect fairness constraints with respect to possibly sensitive features such as age. The DDCAL algorithm can be interpreted as fair clustering technique with respect to the even distribution of the data points over the clusters, but do not assign any sensitivity to features.

## Summary and Outlook

### Summary Regarding Research Questions:

This work addresses research questions RQ1–RQ3 as set out in Section 1 as follows: DDCAL constitutes a heuristic clustering algorithm for evenly distributing data into clusters over a maximum number of low variance clusters based on the iterative feature scaling method min-max normalization which is also known as rescaling (RQ1). Regarding RQ2, we studied DDCAL on several synthetic and real-world data sets and compared the results to 11 existing clustering algorithms. From the synthetic data sets it can be observed that DDCAL performs well for data with outliers and data following (tailed) distributions with one peak, which show a bell curve such as normal and gumbel distribution. Additionally, DDCAL has a good performance on uniformly distributed data, or the number of peaks in the data set is equal or higher than the number of targeted clusters (*M*). If the number of peaks is lower than the number of targeted clusters or the data set to cluster is exponentially distributed, the DDCAL shows weaknesses. For DDCAL, outliers are particularly treated in a way such that they do not “dominate” the resulting clusters. Three use cases from process model/mining to map visualization indicate that DDCAL results in a more differentiated color grading and hence might lead to a more effective visualization of the data (RQ3).

### Discussion:

The assessment of the algorithms is based on four quality metrics applied to synthetic as well as real-world data sets. We do not employ supervised quality measures because no gold standard for the clusters on the used data sets is available which perfectly suits the trade-off between the metrics SED, an even distribution of data points into clusters and the classical clustering metrics SV and MSC. As shown in Section 5, even the definition of clustering is still an open discussion. The usage of unsupervised quality measures is always biased (Handl et al., 2005; Thrun, 2021) and therefore we evaluate the results based on use cases where we show what additional information could be observed by using DDCAL in comparison to other algorithms which had a good performance on the employed quality metrics.

We observed that DDCAL basically outperforms the other algorithms when evenly distributing data over all clusters and shows average results on building low variance clusters. Thus, DDCAL can be seen as good “all-rounder” for use cases demanding for evenly distributing data elements into a given number of clusters. k-means++ yields good results for all quality metrics. Gaussian kernel k-means performs well regarding an even distribution of data over all clusters. Both algorithms have one drawback, which is the missing “reproducibility” of the results because the initial cluster centers are set differently after each initiation run, which leads to different cluster results after every execution. Gaussian kernel k-means has further problems: The algorithm produces overlapping clusters in some cases and has a high time and space complexity. Moreover, for Gaussian kernel k-means and other existing algorithms analyzed in this work requiring input parameters, there is no research for setting these parameters for 1d data to produce results with an even distribution of data over all clusters and at the same time, having a low variance in clusters. We tackled this problem by simulating different input parameters, but possibly better approaches may be used with further research.

### Higher-Dimensional Data:

Currently, we are working on extending DDCAL for clustering multidimensional data sets. Starting with 2d data, we follow two directions using (a) several distance measures and (b) pre-processing the data. For (a), we can use, for example, the Euclidean distance, Manhattan distance, cosine coefficient, Jaccard coefficient, dice coefficient, Minkowski distance, root mean squared error coefficient^14^, and TS-SS (Heidarian and Dinneen, 2016) by comparing them with extreme data points, like (max-x-value/min-y-value), (min-x-value/max-y-value), (max-x-value/max-y-value), and (min-x-value/min-y-value). Option (b) includes an additional pre-processing step which is responsible for converting the multidimensional data points into one dimension and then using the core concepts of DDCAL. Subsequently, we aim at developing an algorithm for merging the produced clusters from each dimension for building multidimensional clusters.

### Future Work:

Beyond data visualization, we will evaluate how DDCAL can be used for problems like clustering test scores of students for grading (Faber, 1994) or to build evenly distributed learning groups based on previous test scores. Additionally, we will investigate whether and how DDCAL can be used for indexing of data to achieve, for example, a faster information retrieval.

## Figures and Tables

**Fig. 9:**
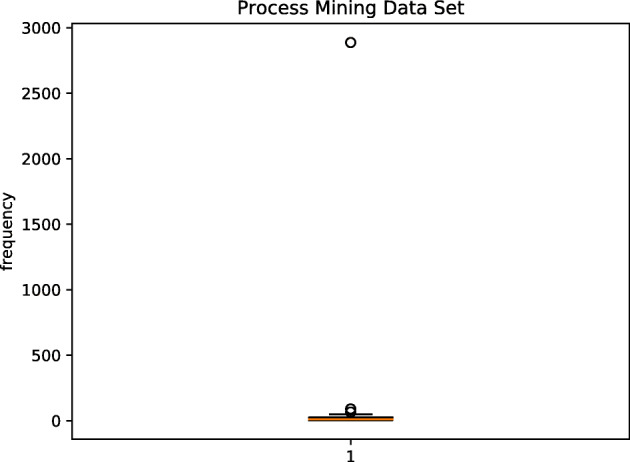
Box plot of Data Set 1

**Fig. 10:**
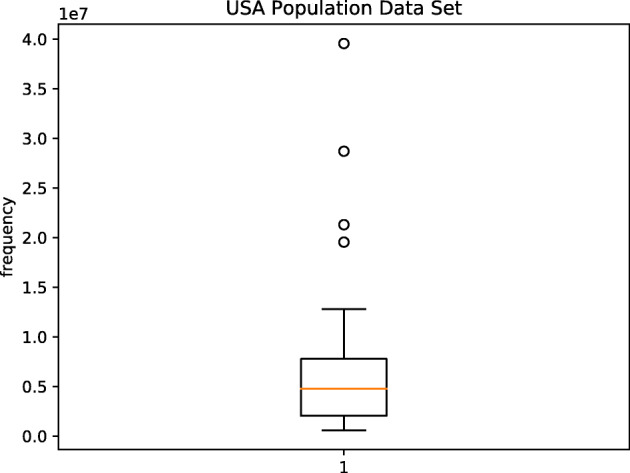
Box plot of Data Set 2

**Fig. 11:**
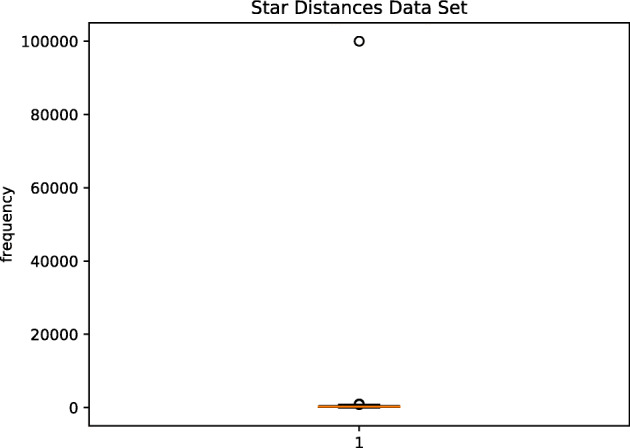
Box plot of Data Set 3

**Fig. 12:**
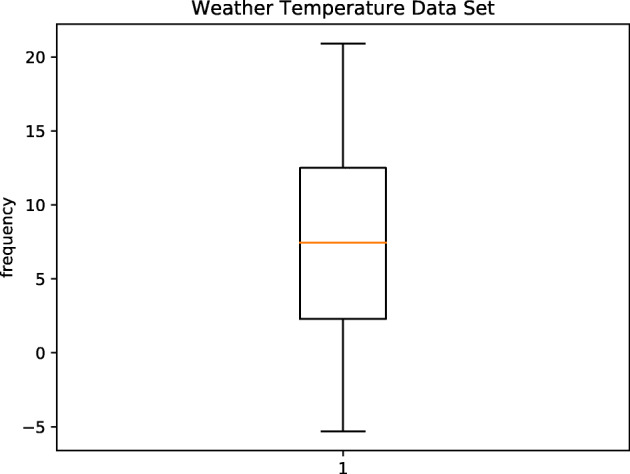
Box plot of Data Set 4

## Data Availability

The generated and analyzed data during assessment of the algorithms are (partly) available in a GitHub repository.^33^

## Appendix 1. Data set descriptions

### Data Set 1 (Process Mining, Search Process Models)

The data set^15^ contains the search frequencies (column freq) of 33 nodes labeled with the corresponding search term from a customer search process (i.e., a directed graph) that was discovered using process mining techniques. For an overview on process mining techniques such as the Heuristic Miner, we refer to van der Aalst (2016). For detailed insights into customer process mining, we refer to Lux et al. (2018) and Bernard and Andritsos (2019).

In general, the representation of process mining results poses a challenge, and representations of process models are denoted as “maps” for process execution behavior for users (van der Aalst & et al, 2011). Specifically, for the analysis of customer behavior, the process model reflects search processes which were mined from event logs collected through the information system.^16^ These logs were generated by tourists through keyword-based entered search terms to find touristic activities in this information system over a period of time. The data contains one outlier reflecting the * search^17^ which accounts for 2887 out of 3444 searches (i.e., 84%). Moreover, the data is skewed, i.e., contains more nodes with small frequencies than nodes with large ones: 16 out of the 33 nodes (i.e., 48%) have a search frequency ≤ 5, reflecting search terms such as hiking tours or sports family. One could argue to remove the * search term from the analysis. However, the * search is part of different search paths in the customer processes, and hence conveys meaningful insights into the search behavior (Lux et al., 2018). Without the outlier, which is responsible for a huge gap, the data is comparable to an exponential distribution. The box plot of the data set is shown in Fig. 9.

### Data Set 2 (United States Population)

The estimated population of the United States (US) per state in 2018 (1 year) is provided from the census data base census.gov of the US government.^18^ Column S0101_C01_001E contains the number of total population per state. We exclude Puerto Rico, Hawaii, and Alaska by deleting the associated rows, as these states are not displayed in our final visualization on the map. We added the population from District of Columbia to the state Maryland and removed the corresponding row, because it is part of the latter state. Also, the row United States, which contains the whole estimated population of the United States, was removed. Thus, the final data set of US population per state covers to 48 states. The data is skewed and comparable to an exponential distribution with many gaps: 25 (52%) of the 48 states, for example, have less than 5 Mio inhabitants where 4 (8%) states have more than 15 Mio inhabitants. The box plot of the data set is shown in Fig. 10.

### Data Set 3 (Stars)

Distances from earth to observed stars are stored in a star database.^19^ Column dist represents the distance information in 119,614 rows. Distances are stored in the unit parsecs, which is the most common unit in astrometry. For conversion of parsecs to light years, they are multiplied by 3.262. A distance ≥= 100,000 indicates that the data is missing or dubious (e.g., negative). Ten thousand two hundred fifteen elements have a distance of 100,000. The data set has therefore two peaks, where the second peak has the same value. There is also a large gap between both peaks. Without these high distance elements, the data set can be seen as heavy tailed and be comparable to a mixture of exponential and Gumbel distribution. Through cluster algorithms like DDCAL, the distances from this data set can be clustered and the number of stars in each cluster can be visualized, for example, to show infographics for the closest, over distant up to the farthest stars away from earth. A corresponding infographics can be found here.^20^ The box plot of the data set is shown in Fig. 11.

### Data Set 4 (Weather)

From the source, there is no description where the data set^21^ originates from and if it is artificially generated or not. However, it seems to be a real-world data set. The data set is stored in the file weather.csv with the column MinTemp and contains 366 elements. We used the lowest temperatures in Celsius over a period of time as input for comparing the algorithms summarized in Section 3.1. The data set follows a uniform distribution. One use case could be to cluster the data into low, medium, and high temperatures and to show the occurrences in an infographics. Alternatively, if more points exist, we could plot a heat map on a geographical map, respectively, a choropleth map. The box plot of the data set is shown in Fig. 12.

### Data Set 5 (Corona Pandemic)

The data set relies on the 2019 Novel Coronavirus COVID-19 (2019-nCoV) Data Repository by Johns Hopkins CSSE.^22^ Particularly, we are interested in the number of confirmed infections, deaths, and recoveries for all countries starting from 22 January 2020.

## Appendix 2.: Implementation of algorithms

DDCAL: Following the pseudo code descriptions of Algorithm 1, the implementation was made in the programming language Python without using additional frameworks, with exception of numpy (version 1.21.2). DDCAL has 6 parameters to set where recommendations on specific data sets are described in Section 3.3. The algorithm produces stable results. The Python implementation of DDCAL is accessible on GitHub.^23^ and usable on PyPI.^24^

kmeans++: The kmeans++ implementation is based on Arthur and Vassilvitskii (2006). We used the Python framework scikit-learn (version 0.24.1) to create the cluster centers. An input parameter is given for the number of aimed cluster centers. Then for each data point, the nearest cluster center is calculated and assigned to this cluster by using the Euclidean distance. The initialization method (k-means++) which selects the initial cluster centers for k-means clustering is performed according to Arthur and Vassilvitskii (2007) and thus is a stochastic algorithm, which may produces different results after each run. We used this method, because it is the most common method for k-means clustering which is used as well as default in the popular Python framework scikit-learn and has two advantages: (a), to avoid k-means for converging to a local minimum and (b), to avoid iterations which saves computing time. However, we also tried “random” as initialization method and the results were very close to the results from the “k-means++” initialization method. E.g., after execution of 10 runs on normal distribution (cf. Section 3.2), we observed, that the SED value was most of the times slightly higher, SV was most of the times nearly the same MSC was always the same (cf. metrics from Section 2.2). Details about further default parameters (e.g., maximum iterations = 300) which were used as parameters in the algorithm are shown in.^25^

Jenks natural breaks: The core idea of the algorithm is based on Jenks natural breaks (Jenks, 1967) and improved in terms of time complexity with the Fisher-Jenks algorithm as described in.^26^ We used the Python framework jenkspy (version 0.2.0) for creating the breaks. Based on the calculated breaks, we created classes and assigned every data point to one of these classes. The algorithm contains one input parameter for defining the number of aimed clusters and aims to reduce the variance within classes and maximizes the variance between classes. It is popular in cartography to generate choropleth maps. The algorithm contains basically three steps^27^, where in the first step, for each data point, the “sum of squared deviations for array means” (SDAM) is calculated. In the second step, for each range combination, the “sum of squared deviations for class means (SDCM_ALL) is calculated. Next, smallest variation within classes is chosen which leads to the third step that assesses the “goodness of variance fit” (GVF := $\frac {SDAM-SCDM}{SDAM}$) which ranges from 1 (perfect fit) to 0 (worst fit). Thus, the best combination has the highest value for GVF and is finally chosen as result. The algorithm produces stable results.

Head/tail breaks: This algorithm is mainly inspired by the Jenks natural breaks algorithm (Jiang, 2013) and creates breaks as well as output. We used an existing Python implementation.^28^ and assigned the data points to classes like explained in Jenks natural breaks. The results of the algorithm are stable. The algorithm contains no input parameter. Thus, the number of envisaged clusters cannot be specified. Basically, the algorithm performs best for visualization of choropleth maps with data sets where far more small data points than large ones exist.

DBSCAN: The core idea of the algorithm is based on Ester et al. (1996). We used the Python framework scikit-learn (version 0.24.1) for creating the clusters. The algorithm produces stable results and has two input parameters. The parameter which is responsible for the minimum number of points was set to 1 which implies that no data points were discarded. The parameter *𝜖* was calculated through simulations for suitable maximum allowed gaps. There exist many methods to calculate an optimum value for *𝜖*, like plotting distances between data points and selecting *𝜖* as the point of the maximum curvature. These approaches did not work in our case because we have a fixed number of envisaged clusters which is not considered in such approaches. Furthermore, the algorithm has no parameter for a number of aimed clusters as upper boundary. Therefore, we implemented a simulation method with the input of different *𝜖* values which identified the maximum score of even distributed data points over all clusters (SED) within the given number maximum clusters (*M*), which were in our case the number of aimed clusters (for further details on SED and M, cf. Section 2.2). The range of *𝜖* values used for simulation was determined by the minimum and maximum gap between data points in a given data set. We used 1000 simulation steps to test evenly spaced *𝜖* values based on the determined range.

KDE: Kernel density estimation is a statistical method to estimate the probability density function of a random variable (Scott, 2015). KDE has many application possibilities, for example on visualization of data, to plot a density curve in place of plotting a histogram. We used the KDE method for clustering in a Python implementation which is described as follows.^29^ First we calculated an evenly spaced interval by using the KDE with the Gaussian kernel. Then, we calculated its relative minima. We sorted the minima descending and took the first (largest) minimum according to the desired number of clusters. Then, we created a list of classes, which represented our clusters, with the minima as splitting points. Finally, we assigned the data points to the list of classes. For the KDE algorithm we used the framework scikit-learn (version 0.24.1) and for calculating the minima we used the framework scipy (version 1.6.2). The KDE has only one input parameter called bandwidth h. Like on DBSCAN, we implemented a simulation method with different input parameters of h which had the goal to identify the maximum score of evenly distributed data points over all clusters (SED) for a given number of maximum clusters (*M*) which is the number of aimed clusters (cf. Section 2.2). Thus, there exists no parameter to define aimed clusters and the number of clusters was identified through simulation of different h values. The range of different h values for simulation was determined, by testing different ranges of h values which produced m clusters. After a matching range was found, we executed on this range 1000 simulation steps and the highest result for SED was kept. The results of the algorithm are stable.

GMM: Gaussian mixture model attempts to find clusters from a mixture of a finite number of Gaussian distributions with unknown parameters (Reynolds, 2009), (VanderPlas, 2016). It is a density estimator like the previously introduced KDE. We used the framework scikit-learn (version 0.24.1) for our Python implementation where the default parameter for EM iterations to perform, was set from 100 to 500, otherwise the default parameters were used.^30^ The framework requires as input parameter the number of Gaussian distributions M, and the clusters are built based on the calculated probabilities from the Gaussian mixture model, where data points are assigned to the most probable distribution. The algorithm is based on expectation maximization (EM), where yielded results depend on a trial (Thrun, 2021) and thus, the algorithm is stochastic.

mean shift: The algorithm is described in Comaniciu and Meer (2002) and for the Python implementation we used the framework scikit-learn (version 0.24.1). The framework is designed to assign each data point to its associated cluster and uses the Flat kernel. The algorithm requires as input parameter a defined quantile to estimate the bandwidth h. Following the same logic on simulating input values, as described on DBSCAN and KDE, we implemented a simulation method for different values of quantiles with the goal to identify the best score of even distributed data points over all clusters (SED) within the given number of maximum clusters (*M*) where further details are on SED and *M*, are discussed in Section 2.2. Thus, the algorithm has basically no parameter for aimed clusters or maximum clusters, but the number of aimed clusters was be determined through simulation by testing quantiles ranging 0.005 to 1. The exact range on each data set of different quantiles for simulation was determined, as described on KDE, by simulating different quantiles which produced about m clusters. On each simulation, we executed 100 simulation steps to test a range of quantiles. The algorithm produces stable results.

largest gaps: This algorithm is not based on literature and implemented in a Python framework. It is an ordinary method based on the largest gaps in a sorted data set between adjacent data points for clustering which was implemented as follows: First we sorted the list of data points in ascending order. Then, we calculated and stored for every data point (a) the gap between the current and the previous data point and (b) the average value between the current and the previous data point. After that, we sorted the list of data points based on their calculated gaps (a) in ascending order. The algorithm had only one input parameter which was the number of aimed clusters (*M*). Based on the set number of aimed clusters, we took the number first elements from the list of gaps (a) and stored for every data point the average values (b) in a separate list. This list was finally our classification list for assigning each data points to clusters. For example the first cluster, which was the cluster containing the lowest data points, ranges from the minimum data point to the lowest element of the classification list. The second cluster ranges from the lowest element of the classification list plus one, up to the second lowest element of the classification list, and so on. This algorithm is very intuitive and simple to implement which produces stable results.

Gaussian Kernel k-means: This algorithm is a variant of k-means which was introduced before, but uses the the Gaussian kernel method as non-linear distance function. Therefore, it is a stochastic algorithm, which may produces different results after each run. Because there exists no implementation in the Python Package Index, we used an implementation.^31^ which is based on Dhillon et al. (2004). The implementation was extended by us for handling empty clusters on an iteration by terminating the algorithm if an empty cluster was found. The algorithm has three input parameters. The first parameter defines the number of aimed clusters (*M*), the second parameter defines the initial positions of the centroids, which was set to “random” and the third parameter defines the variance of the kernel. The latter parameter was optimized by using a simulation method, as described in DBSCAN, KDE, and mean shift. As mentioned on KDE and following this approach, the range of different variance values for simulation was determined by testing variances, on which the results produced m clusters. With exception of Data Set 1, where we tested variances ranging from 10,000 to 1,000,000, the range for simulation of the variances was 0.1 to 30 by using 100 simulation steps. Because the initial positions of the centroids were chosen randomly (second parameter as mentioned before), the simulation method was extended to simulate each variance value 10 times which keeps the best result with the maximum score for even distributed data points over all clusters (cf. metric SED from Section 2.2). Thus, on each range, 1000 simulation steps were executed in total and the result containing the highest SED value was kept. The algorithm has two major drawbacks: (a) it was the only one which produced overlapping cluster ranges. Such results were filtered out through a python implementation which extended the simulation method. (b) The time and space complexity of O(*n*^2^) (Sarma et al., 2013) resulted in long execution times on “huge” data sets (containing > 1000 data points).

k-medoids: The algorithm is another variant of k-means which was introduced before. Instead of calculating centroids like in k-means which may no be actual data points in a data set, where they are the average between the points in the cluster, k-medoids chooses always actual data points as centers, which are called “medoids” (Park and Jun, 2009). Through the minimizing of the sum of pairwise dissimilarities instead of minimizing the sum of squared Euclidean distances, the algorithm is more robust to noise and outliers than k-means. In contrast to k-means which requires generally the Euclidean distance as dissimilarity measure, k-medoids can be used with arbitrary dissimilarity measures for producing effective solutions, where we used for example the Manhattan distance. For the Python implementation, we used the framework scikit-learn-extra (version 0.2.0) with the dissimilarity metric Manhattan and the initialization method k-medoids++. Because of the random initialization method, the algorithm is, like k-means++, a stochastic algorithm, which may produce different results after each run.

Trimmed k-means: It is a variant of k-means which optimizes the algorithm under trimming a portion of the data points in a data set. The algorithm has the aim of robustifying k-means (Cuesta-Albertos et al., 1997). For implementation of the algorithm used, the trimcluster packages from R, which was called from Python where the analysis method of the results was performed. We used this particular R package, and not a python framework, because there was no implementation of trimmed k-means in the Python Package Index. The trim factor was set to 0.1 which means that 10% of outliers are detected by the algorithm from the data to cluster and put in an separate “outlier” cluster. The number of algorithm runs from initial means, which are randomly chosen from the data points, was set to 500. The rest of the parameters were set to their default parameters, as described in.^32^

## Footnotes

1 https://www.statista.com/statistics/871513/worldwide-data-created/, accessed 2022-08-08

2 https://fluxicon.com/disco/, accessed 2022-08-08

3 https://geographicdata.science/book, accessed 2022-08-08

4 https://github.com/luxmar/DDCAL

5 https://github.com/luxmar/DDCAL/tree/main/tests/data

6 https://github.com/luxmar/DDCAL/tree/main/supplemental

7 https://github.com/luxmar/DDCAL/tree/main/supplemental

8 https://github.com/luxmar/DDCAL/tree/main/supplemental

9 https://github.com/luxmar/DDCAL/tree/main/supplemental

10 https://scikit-learn.org/stable/modules/generated/sklearn.cluster.KMeans.html, accessed 2022-08-08

11 https://tinyurl.com/tgw4vty, accessed 2022-08-08

12 https://covid19.who.int, accessed 2022-08-08

13 https://corona.swis.io/

14 https://developers.google.com/machine-learning/clustering/similarity/manual-similarity, accessed 2022-08-08
